# Migratory birds as disseminators of ticks and the tick-borne pathogens *Borrelia* bacteria and tick-borne encephalitis (TBE) virus: a seasonal study at Ottenby Bird Observatory in South-eastern Sweden

**DOI:** 10.1186/s13071-020-04493-5

**Published:** 2020-12-03

**Authors:** Peter Wilhelmsson, Thomas G. T. Jaenson, Björn Olsen, Jonas Waldenström, Per-Eric Lindgren

**Affiliations:** 1 Division of Inflammation and Infection, Department of Biomedical and Clinical Sciences, Linköping University, Linköping, Sweden; 2 Department of Clinical Microbiology, Region Jönköping County, Jönköping, Sweden; 3 Department of Organismal Biology, Evolutionary Biology Centre, Uppsala University, Uppsala, Sweden; 4 Zoonosis Science Center, Department of Medical Sciences, Uppsala University, Uppsala, Sweden; 5 Center for Ecology and Evolution in Microbial Model Systems, Linnaeus University, Kalmar, Sweden

**Keywords:** Migratory birds, *Borrelia* prevalence, *Borrelia burgdorferi* (*sensu lato*), *Borrelia miyamotoi*, Tick-borne encephalitis virus

## Abstract

**Background:**

Birds can act as reservoirs of tick-borne pathogens and can also disperse pathogen-containing ticks to both nearby and remote localities. The aims of this study were to estimate tick infestation patterns on migratory birds and the prevalence of different *Borrelia* species and tick-borne encephalitis virus (TBEV) in ticks removed from birds in south-eastern Sweden.

**Methods:**

Ticks were collected from resident and migratory birds captured at the Ottenby Bird Observatory, Öland, Sweden, from March to November 2009. Ticks were molecularly identified to species, and morphologically to developmental stage, and the presence of *Borrelia* bacteria and TBEV was determined by quantitative real-time PCR.

**Results:**

A total of 1339 ticks in the genera *Haemaphysalis, Hyalomma*, and *Ixodes* was recorded of which *I. ricinus* was the most abundant species. Important tick hosts were the European robin (*Erithacus rubecula*), Blackbird (*Turdus merula*), Tree pipit (*Anthus trivialis*), Eurasian wren (*Troglodytes troglodytes*)*,* Common redstart (*Phoenicurus phoenicurus*)*,* Willow warbler (*Phylloscopus trochilus*), and Common whitethroat (*Sylvia communis*). *Borrelia* bacteria were detected in 25% (285/1,124) of the detached ticks available for analysis. Seven *Borrelia* species (*B. afzelii*, *B. burgdorferi* (*s.s.*), *B. garinii*, *B. lusitaniae*, *B. turdi, B. valaisiana*, and *B. miyamotoi*) were identified. *B. turdi* was recorded for the first time in ticks in Sweden. The number of *Borrelia* cells per tick ranged from 2.0 × 10^0^ to 7.0 × 10^5^. *B. miyamotoi*-containing ticks contained a significantly higher median number of *Borrelia* cells than *B. burgdorferi* (*s.l.*)-containing ticks. *B. garinii* and *B. miyamotoi* were the most prevalent *Borrelia* species in tick larvae. Larvae of *I. ricinus* with *B. garinii* were removed from seven bird species, particularly *S. communis* and *A. trivialis*, which may suggest that the larvae had contracted the *Borrelia* bacteria from or via these birds. Also, a high percentage of tick larvae containing *B. miyamotoi* was removed from *E. rubecula.* All ticks were negative for TBEV.

**Conclusions:**

The results corroborate the view that the contributions of birds to human disease are substantial, particularly as blood hosts for ticks and for their short-, medium-, and long-distance dispersal. Moreover, several ground-foraging bird species appear to be important for the maintenance and dispersal of *Borrelia* species. The absence of TBEV in the ticks conforms to other similar studies.
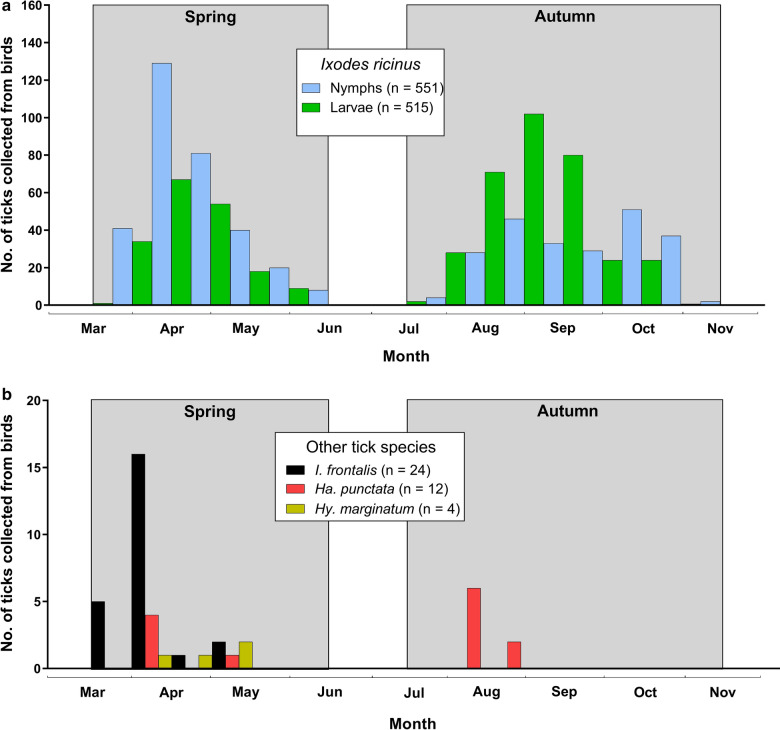

## Background

Several species of ticks are known to infest birds, and such birds can disperse ticks over short, medium and long distances [1]. Certain potentially human-pathogenic microorganisms are vectored by ticks. Some of these microorganisms may be harboured in both ticks and birds. Obviously, microorganism-containing vector-competent ticks, while feeding on a susceptible avian host, can transmit the microorganism to its host. Certainly, transmission of a blood-borne microorganism can also take place in the opposite direction, whereby infectious, transmission-competent birds (transmission hosts or reservoir-competent hosts) can infect feeding, susceptible ticks. During their flights birds can act as “vehicles” for the geographic spread of different types of human-pathogenic microorganisms, i.e. for microorganisms which are maintained in both birds and ticks, for microorganisms mainly or only present in ticks, and for microorganisms mainly or only present in birds. Tick-associated microorganisms, with proven, suspected, or unknown human pathogenicity which have been detected in bird-infesting ticks in Europe include *Borrelia afzelii* [2–5], *Borrelia burgdorferi* (*s.s*) [2, 4, 6, 7], *Borrelia garinii* [2–5, 7, 8], *Borrelia lusitaniae* [9], *Borrelia turdi* [3, 5, 10–12], *Borrelia valaisiana* [2–4, 10, 11, 13], *Borrelia spielmanii* [4, 5], *Borrelia miyamotoi* [2, 4, 14], *Neoehrlichia mikurensis* [4, 14–16], *Rickettsia aeschlimannii* [16, 17], *Rickettsia africae* [17], *Rickettsia helvetica* [14, 16, 18, 19], *Rickettsia japonica* [16], *Rickettsia monacensis* [16], *Anaplasma phagocytophilum* [14, 19], *Babesia venatorum* [16], *Babesia microti* [16], tick-borne encephalitis virus (TBEV) [16, 20], Alkhurma haemorrhagic fever virus (AHFV) [21], and Crimean-Congo haemorrhagic fever virus (CCHFV) [22].

Seasonal migration is common among birds breeding in northern latitudes but shows large variation between and within species in timing and direction of migration as well as distance travelled. Most migratory birds divide the trip into several migration legs, interspersed with time spent on stopover sites along the route. At suitable stopover sites, birds feed and prepare for the next leg of migration, providing opportunities for new ticks to attach and already fed ticks to detach from the birds, thereby following birds along the migratory routes and potentially creating new foci of tick-borne diseases.

The overall aim of this study is to better understand the potential role of birds as disseminators of ticks and tick-borne pathogens into Sweden during their northward, spring, and early summer migration and from Sweden during their southward, late summer and autumn migration. In particular, the present investigation was designed to determine the abundance of different tick species and tick stages infesting migratory and other birds captured at one of Sweden’s most important bird observatories. Moreover, we aimed at determining the prevalence of *Borrelia* and TBEV contents in the ticks infesting the birds.

## Methods

### Description of the sampling site, Ottenby Bird Observatory

Ottenby Bird Observatory is located on the southern point of the island of Öland in south-east Sweden. At the observatory, migratory birds have been banded since 1946, and more than 1 million birds have been caught, providing a solid knowledge base on which bird populations that pass through the area [23, 24]. Apart from funnelling migratory birds during migration, the area also functions as stopover site and breeding ground for many species of birds. In spring, the vast majority of birds caught at the observatory have just arrived after a night of migration, including passage over stretches of open water (the Baltic Sea), while in autumn, birds caught will be a mixture of birds arriving from areas to the north and east, and those that have spent some time on stopover in the area and restarting migration. The reserve in which the observatory is situated encompasses an area of about 25 km^2^ with shore meadows, pastures, and woodland dominated by stands of old oaks (*Quercus robur*) and birch (*Betula* sp.). The area is grazed by cattle (*Bos taurus*), sheep (*Ovis aries*) and fallow deer (*Dama dama*).

### Bird captures and tick collections

Sampling of birds was approved by the Swedish Board of Agriculture, delegated through the Animal Research Ethics Committee in Linköping (decision 43–09). Birds were captured during the general trapping activities of staff members at the Ottenby Bird Observatory (56° 12′ N, 16° 24′ E). With the approval of the Swedish Museum of Natural History in Stockholm, Sweden, mist nets and Heligoland traps were used to capture birds during the periods 15 March–15 June and 15 July–15 November 2009 as part of the observatory’s standardized trapping scheme. Trapped birds were banded, measured, and identified to species level and, when possible, sexed and aged. At the observatory, trapping starts half an hour before sunrise and ends at 11 a.m., which means that the length of the trapping day varies with season. To achieve a representative sample of birds for this study, and to comport with other duties at the observatory, we devised a schedule wherein every second day each bird captured during the first four working hours (start 30 min before sunrise) was checked for ticks around the base of the beak and around the eyes and ears, and every other day each bird captured during the last four working hours (7–11 a.m.) was checked. Ticks were removed with forceps and photographed as described below. Ticks were stored individually in empty snap-lid tubes at − 70 °C.

### Classification of bird migration categories

As noted above, the majority of birds screened for ticks in this study were caught during migration. We broadly classify the investigated species into residents, short-distance migrants, partial migrants, and long-distance migrants based on band recovery data from the observatory and Sweden as a whole [25]. The resident bird species category comprises four species—*Passer domesticus*, *Passer montanus*, *Corvus monedula*, and *Pica pica*–that are sedentary in the area and show little to no migratory propensity apart from local dispersal. The division between short- and long-distance migrants is based on distance between breeding areas and non-breeding areas, where species that mainly winter in Europe are classified as short-distance migrants and species wintering predominantly in Africa or Asia as long-distance migrants. A special category is the partial migrants, a category which here is used to denote species of which some individuals are residents, some are short-distance migrants, and some alternate between these categories depending on the severity of winter or other environmental cues. Thus, the categories used here reflect the populations of the species studied at Ottenby and would in some instance be categorized differently at other study locations in the distribution range.

### Determination of developmental stage and species of the ticks

Each tick was photographed dorsally and ventrally using a digital USB microscope (Dino-Lite Long AM4013TL, AnMo Electronics Corp., Taiwan), as previously described [15]. Based on the photographs, each tick was morphologically identified to developmental stage (larva, nymph, or adult) and sex of adults. To identify the genus and species of the ticks, each specimen was analysed using molecular methods. All ticks were analysed by a PCR method targeting the tick mitochondrial 16S rRNA gene, as previously described [26].

### Total nucleic acid extraction and cDNA synthesis from ticks

Collected ticks were homogenized individually by bead beating in 2 ml safe-lock microcentrifuge tubes (Eppendorf AG, Hamburg, Germany) with a 5-mm stainless steel bead (Qiagen, Hilden, Germany) in 350 μl RLT buffer (Qiagen), supplemented with 1% 2-mercaptoethanol (Sigma-Aldrich, Stockholm, Sweden) using a TissueLyser II (Qiagen) for 2 min at 25 Hz. After centrifugation at 20,000×*g* for 3 min, 300 μl supernatant was transferred to new microcentrifuge tubes for total nucleic acid (NA) extraction, using MagAttract® Viral RNA M48 kit (Qiagen) in a BioRobot M48 workstation (Qiagen), using a 65 μl elution volume. Each batch of 24 samples consisted of 22 ticks, one positive control [5 μl of *B. burgdorferi* (*s.s.*) B31 ATCC 35210 (10^8^ cells/ml) and 5 μl inactivated TBEV strain K23, Encepur®, Chiron Vaccines, Marburg, Germany] and one negative control (H_2_O) that were extracted simultaneously. The eluted NA was reverse-transcribed to cDNA using illustra™ Ready-to-Go RT-PCR Beads kit (GE Healthcare, Amersham Place, UK). Twenty microlitres NA and 10 μl pd(N)6 random hexamer primers (0.25 μg/μl) were incubated for 5 min at 97 °C and then mixed with one RT-PCR bead dissolved in 20 μl RNase-free water. The mixture was incubated for 30 min at 42 °C, followed by 5 min at 97 °C, producing 50 μl cDNA. Pipetting was performed using a CAS1200 pipetting robot (Corbett Robotics Inc., San Francisco, CA) and incubation using a PTC 100 thermal cycler (M.J. Research, Inc., Waltham, MA).

### Detection and quantification of Borrelia bacteria and TBEV by real-time PCR analyses

Two microlitres of cDNA per reaction was used in a LUX™ real-time PCR assay to detect and quantify a 131-bp-long fragment of the *Borrelia* spp. *16S* rRNA gene, using genus-specific primers and a serial dilution of plasmid standard [27]. Two microlitres of cDNA per reaction was used in a multiplex TaqMan™ real-time PCR assay to detect and quantify all three subtypes of TBEV, using primers, probes, and a serial dilution of plasmid standard [28]. PCR reagents and templates were added to a 96-well real-time PCR plate by using a CAS1200 pipetting robot (Corbett Robotics Inc.). The real-time PCR analyses were carried out using the BioRad CFX96 Real-Time PCR Detection System (Bio-Rad Laboratories, Hercules, CA).

### Determination of *Borrelia* species by conventional PCR and nucleotide sequencing

To determine *Borrelia burgdorferi* (*s.s*) species of the samples positive in the LUX real-time PCR assay, a nested conventional PCR assay using primers targeting the intergenic spacer region (IGS) between *5S* and *23S* rRNA genes was applied, as described in [29]. Samples that failed to produce PCR products with this assay were instead analysed with primers targeting the IGS between *16S* and *23S* rRNA genes to determine other *Borrelia* spp., as described in [30, 31]. Tick samples, positive for *Borrelia* spp. in the LUX real-time PCR assay, which failed to produce PCR products with the 5S-23S IGS assay and 16S-23S IGS assay, were denoted ‘untypeable’.

Sequencing of all the PCR products was performed by Macrogen Inc. (Amsterdam, The Netherlands). Chromatograms were initially edited using BioEdit software v7.0 (Tom Hall, Ibis Therapeutics, Carlsbad, CA) and sequences were examined using Basic Local Alignment Tool (BLAST). The appearance of dual peaks in chromatograms was interpreted as at least two *Borrelia* species in the sample and thus denoted as ‘mixed’. An additional file shows the aligned sequences (see Additional file 1).

### Statistical analyses

Data were presented as medians with interquartile range (IQR) for numerical variables and as percentage for categorical variables. The numerical variables (i.e. the number of *Borrelia* spp. cells of different *Borrelia* spp. detected in the ticks) were analysed using Kruskal-Wallis test, which, if significant, was followed by Dunn’s multiple comparison test. The categorical variables (e.g. developmental stages of the tick; tick species; spring vs. autumn, etc.) were analysed using chi-square test, but when the expected frequency was < 5 in at least one of the cells of the contingency table, Fisher’s exact test with a confidence interval (CI) of 95% was used instead. Statistical analyses were performed, and graphs and figures were drawn using GraphPad Prism version 8.0.0 for Windows (GraphPad Software, San Diego, CA). *P*-values ≤ 0.05 were considered statistically significant.

## Results

### Ticks collected from birds and seasonal dynamics of infestation pattern

**Fig. 1:**
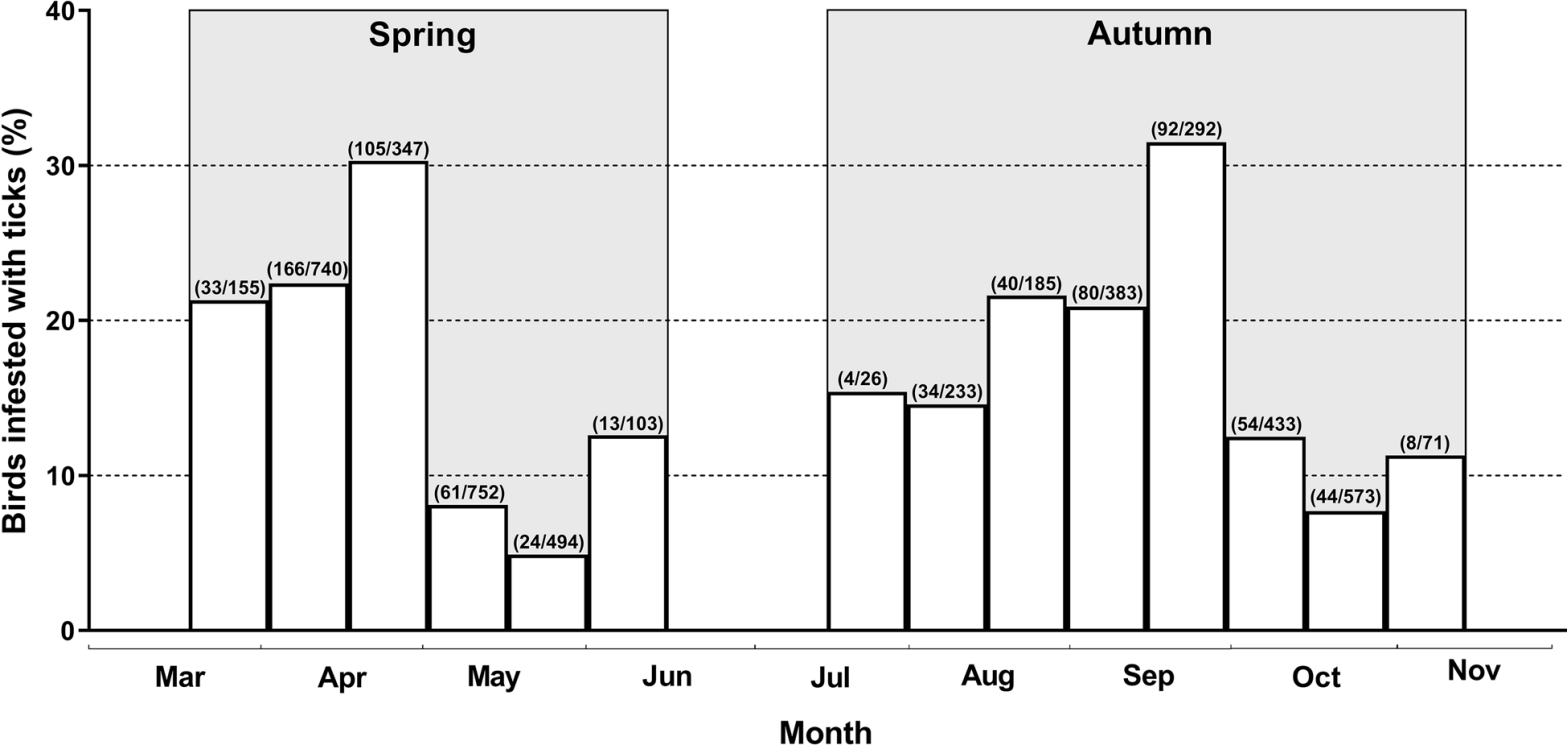
Monthly patterns of birds infested with ticks during the study period. In brackets: number of bird captures infested by ticks/total number of bird captures checked for ticks during the collection period (Spring: 15 March–15 June; Autumn: 15 July–15 November 2009, shaded in gray). One bird capture was excluded from the analysis because of missing collection data

A total of 4601 bird individuals (4788 bird captures) of 65 species was examined for ticks at least once during the study period, i.e. 15 March–15 June and 15 July–15 November 2009, at the Ottenby Bird Observatory. A total of 749 bird individuals (759 bird captures) of 35 species was infested with at least one tick specimen (Table 1). Calculations based on bird captures showed that the monthly prevalence of birds infested with one or more ticks during the study period ranged from 4.90 to 31.5%, with the lowest and highest infestation prevalence in the second half of May and second half of September, respectively (Fig. 1).

**Table 1:** Bird species with tick infestation patterns, collected at the Ottenby Bird Observatory, Sweden, 2009

Bird species	Migratory category^a^	No. of bird captures	No. of infested birds (%)	No. of ticks	Mean no. ticks per infested bird ± SE	Median no. ticks per infested bird (IQR)	Tick species and developmental stage^c^					
							*Ixodes* spp.	*I. r*	*I. f*	*H. p*	*H. m*	**ND**
*Accipiter nisus*	PM	5	1 (20)	1	–	–		1N				
*Acrocephalus palustris*	LM	21	3 (14)	4	1.3 ± 0.33	1 (1.00–2.00)		4N				
*Acrocephalus scirpaceus*	LM	11	1 (9.0)	1	–	–		1N				
*Anthus trivialis*	LM	39	26 (67)	93	3.6 ± 0.73	2 (1.00–4.25)	4L	52L, 33N				4
*Carduelis cannabina*	SM	15	1 (6.7)	1	–	–				1L		
*Carduelis flammea*	SM	36	2 (5.6)	7	3.5 ± 1.50	3.5		7N				
*Certhia familiaris*	PM	26	2 (7.7)	4	2.0 ± 0.00	2		2L				2
*Cyanistes caeruleus*	PM	58	4 (6.9)	7	1.6 ± 0.75	1 (1.00–3.25)		1N		4L		2
*Emberiza schoeniclus*	SM	28	2 (7.1)	3	1.5 ± 0.50	1.5		2N				1
*Erithacus rubecula*	SM	1551	368 (24)	619	1.7 ± 0.09	1 (1.00–2.00)	38L, 13N	278L, 194N, 3U	5L, 11N, 1A		1L	75
*Fringilla coelebs*	SM	37	6 (16)	8	1.3 ± 0.21	1 (1.00–2.00)		4L, 2N			1A	1
*Fringilla montifringilla*	SM	8	1 (13)	1	–	–		1L				
*Hippolais icterina*	LM	50	2 (4.0)	2	1.0 ± 0.00	2		1L, 1N				
*Lanius collurio*	LM	43	3 (7.0)	3	1.0 ± 0.00	1 (1.00–1.00)		3N				
*Luscinia luscinia*	LM	18	10 (56)	29	2.9 ± 0.78	2 (1.75–3.25)		20L, 9N				
*Luscinia svecica*	LM	28	2 (7.1)	2	1.0 ± 0.00	1	1N	1N				
*Motacilla alba*	LM	23	1 (4.3)	3	–	–		2L		1L		
*Oenanthe oenanthe*	LM	6	1 (17)	1	–	–				1L		
*Parus major*	PM	44	3 (6.8)	8	2.7 ± 1.67	1 (1.00–6.00)		2N				6
*Passer domesticus*	R	16	1 (6.3)	1	–	–		1N				
*Phoenicurus ochruros*	LM	10	2 (20)	2	1.0 ± 0.00	1		2N				
*Phoenicurus phoenicurus*	LM	134	40 (30)	64	1.6 ± 0.22	1 (1.00–2.00)		29L, 32N				3
*Phylloscopus collybita*	SM	79	3 (3.8)	3	1.0 ± 0.00	1 (1.00–1.00)		2N				1
*Phylloscopus trochilus*	LM	810	38 (4.7)	57	1.5 ± 0.14	1 (1.00–2.00)	1L, 1N	19L, 30N	1L		2N	3
*Prunella modularis*	SM	44	16 (36)	24	1.5 ± 0.14	1 (1.00–1.00)	2N	3L, 19N				
*Regulus regulus*	PM	319	9 (2.8)	9	1.0 ± 0.00	1 (1.00–1.00)		2L, 7N				
*Sturnus vulgaris*	SM	14	3 (21)	3	1.0 ± 0.00	1 (1.00–1.00)		2N		1L		
*Sylvia atricapilla*	SM	58	4 (6.9)	4	1.0 ± 0.00	1 (1.00–1.00)		2L, 1N				1
*Sylvia communis*	LM	123	27 (22)	49	1.8 ± 0.37	1 (1.00–2.00)		23L, 17N		4L		5
*Sylvia curruca*	LM	270	10 (3.7)	11	1.1 ± 0.10	1 (1.00–1.00)		7L, 2N				2
*Troglodytes troglodytes*	PM	254	72 (28)	120	1.7 ± 0.20	1 (1.00–2.00)	13L, 2N	59L, 31N, 1U	1L			13
*Turdus iliacus*	SM	29	9 (31)	23	2.6 ± 0.69	2 (1.00–4.50)		1L, 18N	1N			3
*Turdus merula*	PM	147	66 (45)	149	2.3 ± 0.25	1 (1.00–3.00)	6N	8L, 108N, 1U	1L, 2N			23
*Turdus philomelos*	SM	60	9 (15)	22	2.4 ± 0.58	2 (1.00–3.50)	2N	2L, 17N	1N			
Unknown^b^		1	1 (100)	1	–	–		1N				
*Acrocephalus arundinaceus*	LM	1	0	0								
*Acrocephalus schoenobaenus*	LM	23	0	0								
*Anthus pratensis*	SM	5	0	0								
*Carduelis carduelis*	SM	15	0	0								
*Chloris chloris*	SM	16	0	0								
*Carduelis spinus*	SM	9	0	0								
*Carpodacus erythrinus*	LM	2	0	0								
*Coccothraustes coccothraustes*	PM	1	0	0								
*Corvus monedula*	R	1	0	0								
*Delichon urbica*	LM	43	0	0								
*Dendrocopus major*	PM	1	0	0								
*Emberiza citrinella*	SM	23	0	0								
*Ficedula albicollis*	LM	2	0	0								
*Ficedula hypoleuca*	LM	31	0	0								
*Ficedula parva*	LM	8	0	0								
*Hirundo rustica*	LM	25	0	0								
*Jynx torquilla*	LM	1	0	0								
*Locustella naevia*	LM	1	0	0								
*Motacilla flava*	LM	2	0	0								
*Muscicapa striata*	LM	55	0	0								
*Passer montanus*	R	37	0	0								
*Phylloscopus borealis*	LM	1	0	0								
*Phylloscopus sibilatrix*	LM	19	0	0								
*Picus viridis*	R	1	0	0								
*Pyrrhula pyrrhula*	PM	2	0	0								
*Saxicola rubetra*	LM	1	0	0								
*Serinus serinus*	SM	2	0	0								
*Sylvia borin*	LM	42	0	0								
*Sylvia nisoria*	LM	1	0	0								
*Turdus pilaris*	SM	1	0	0								
*Turdus viscivorus*	SM	1	0	0								
Total number of specimens		4788	749	1339	–		56L, 27N	515L, 551N, 5U,	8L, 15N, 1A,	12L	1L, 2N, 1A	145

^a^LM: long-distance migrants (in many cases wintering in Africa); SM: short-distance migrants where the majority of individuals winter in Europe; PM: partial migrants where individuals either migrate short distances within Europe or remain resident; R: residents

^b^Probably *Lanius collurio*

^c^Abbreviations: L, larva; N, nymph; A, adult female; U, undetermined developmental stage due to lack of photos; *I. r*, *Ixodes ricinus*; *I. f*, *Ixodes frontalis*; *H. p, Haemaphysalis punctata; H. m, Hyalomma marginatum*; ND, not determined to genus, species or developmental stage because of lack of photos of ticks and/or loss of sample; SE, standard error; IQR, interquartile range

A total of 1339 ticks was collected from the infested birds. Due to missing photos of ticks and because some ticks were damaged when removed from their hosts, or lost in the nucleic acid extraction process, the genus, species, or developmental stage could not be determined in 10.8% (*n* = 145) of the ticks (Table 1).

Of the remaining ticks, for which the genus, species and/or developmental stage could be determined (*n* = 1194), 1.00% were *Haemaphysalis punctata* (*n* = 12; all larvae), 0.30% were *Hyalomma marginatum* (*n* = 4; 1 larva, 2 nymphs, 1 adult female), and 98.7% were *Ixodes* (*n* = 1178; 579 larvae, 593 nymphs, 1 adult female, and 5 ticks that could not be determined to developmental stage; Table 1). Among the *Ixodes* ticks, 93.0% (*n* = 1095) were identified to species of which 97.8% were *I. ricinus* (*n* = 1071; 515 larvae, 551 nymphs, 5 ticks that could not be determined to developmental stage), and 2.20% were *I. frontalis* (*n* = 24; 8 larvae, 15 nymphs, 1 adult female). Among all ticks of which the developmental stage could be determined, 49.8% were larvae (*n* = 592), 50.0% were nymphs (*n* = 595), and 0.20% were adult females (*n* = 2).

**Fig. 2:**
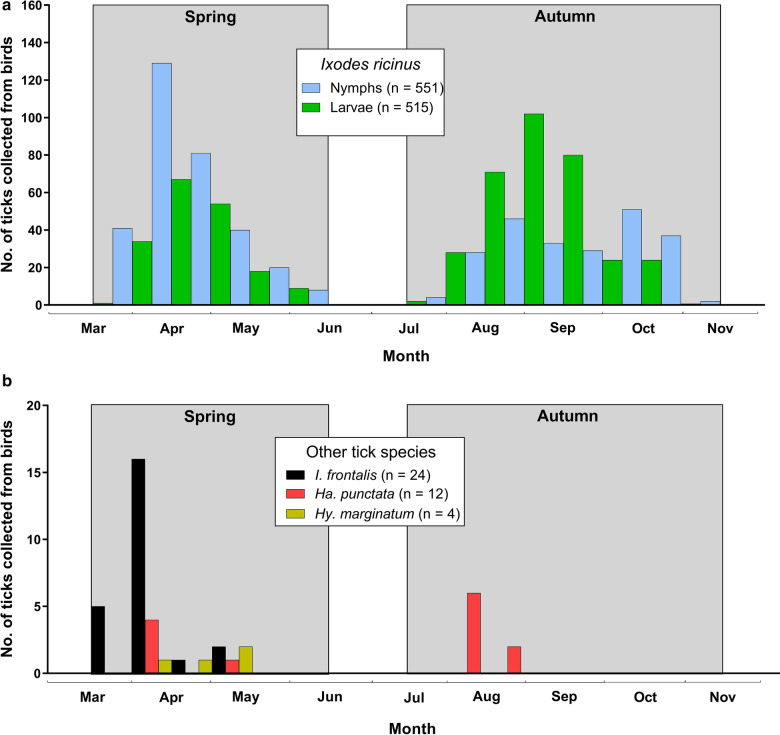
Temporal distribution of ticks removed from birds. Temporal distribution of **a** *Ixodes ricinus* and **b** all other tick species removed from birds during the collection period (spring: 15 March–15 June; autumn: 15 July–15 November 2009, shaded in gray)

Larvae and nymphs of the most prevalent tick species, i.e. *I. ricinus*, were collected during the whole collection period but mainly from mid-March to mid-May and from early August to mid-October (Fig. 2a). Of all collected *I. ricinus* ticks, larvae were more prevalent in autumn (59.0%, 15 July–15 November) than in spring [36.%, 15 March–15 June, (χ^2^ = 53.93, *df* = 1, *P* < 0.0001]. *I. frontalis* and *H. marginatum* were only recorded on birds during the spring collection. *H. punctata* was recorded in both April–May and August (Fig. 2b).

**Fig. 3:**
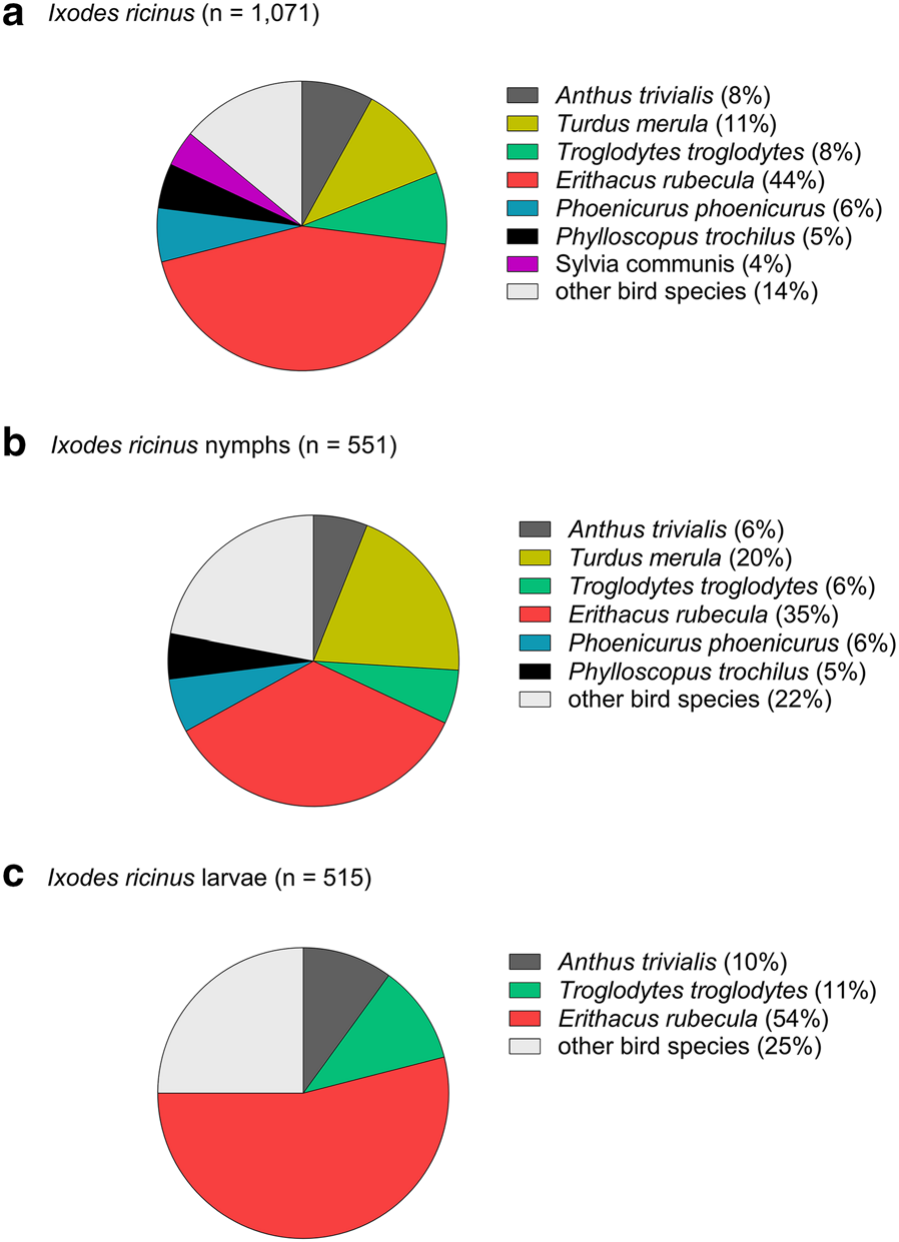
Relative importance of different bird species as hosts for *Ixodes ricinus*. **a** All *I. ricinus* specimen, **b** *I. ricinus* nymphs, and **c** *I. ricinus* larvae. Bird species from which < 30 ticks were collected were included as “other bird species”

Most ticks (46%) were found on the European robin (*Erithacus rubecula*), which was also infested with the highest number of tick species (*n* = 4) (Table 1). Among all ticks identified to species, *I. ricinus* infested the broadest range of bird species, i.e. 32 species (Table 1; Fig 3a). Most of the *I. ricinus* nymphs were removed from *E. rubecula* and Common blackbird (*Turdus merula*) (Table 1; Fig. 3b). Most *I. ricinus* larvae were removed from *E. rubecula*, Eurasian wren (*Troglodytes troglodytes*), and Tree pipit (*Anthus trivialis*) (Fig. 3c).

*I. frontalis* and *H. punctata* were each present on six species of birds (Table 1) and *H. marginatum* on three bird species, i.e. *E. rubecula*, Willow warbler (*Phylloscopus trochilus*), and Common chaffinch (*Fringilla coelebs*).

### Prevalence of *Borrelia* bacteria and TBEV in the ticks

Of the ticks for which the genus, species, or developmental stage could be determined (*n* = 1194), 94.1% (*n* = 1124) were available for analyses of contents of *Borrelia* bacteria and TBEV.

Of all ticks analysed, 25.4% (285/1,124) were positive in real-time PCR for *Borrelia* spp. (Table 2). All 1124 ticks were negative in real-time PCR for TBEV. A significantly higher proportion of nymphs (36.2%, 206/569) than larvae (14.5%, 79/546 [χ^2^ = 69.18, *df* = 1, *P* < 0.0001]) was positive for *Borrelia* spp. Both adult tick females, i.e. one *H. marginatum* and one *I. frontalis*, were *Borrelia* spp. negative.

**Table 2:** Prevalence of *Borrelia* species in ticks removed from bird species captured at Ottenby Bird Observatory, Sweden 2009

Tick species	Stage^a^	No. examined^b^ ticks of each stage	No. (%) positive ticks^c^	No. (%) ticks containing *Borrelia* species determined by nucleotide sequencing^d^														
				*B. a*	*B. g*		*B. v*		*B. b*		*B. t*		*B. l*	*B. m*			Mixed	UT
*I. ricinus*	L	514	80 (16)	1 (1)		19 (24)	3 (4)	1 (1)							8 (10)	2 (2)		46 (58)
	N	549	204 (37)	29 (14)		30 (15)	38 (19)	1 (1)		2 (1)		1 (1)			4 (2)	5 (2)		94 (46)
	U	5	1 (20)				1 (100)											
*I. frontalis*	L	8	0 (0)															
	N	15	3 (20)															3 (100)
	A	1	0 (0)															
*H. punctata*	L	12	1 (8)															1 (100)
*H. marginatum*	L	1	0 (0)															
	N	2	0 (0)															
	A	1	0 (0)															
*Ixodes* spp.	L	11	0 (0)															
	N	5	1 (20)															1 (100)
All species	L	546	81 (15)	1 (1)		19 (23)	3 (4)	1 (1)							8 (10)	2 (3)		47 (58)
	N	571	208 (36)	29 (14)		30 (14)	38 (18)	1 (1)		2 (1)		1 (1)			4 (2)	5 (2)		98 (47)
	A	2	0 (0)															
	U	5	1 (20)				1 (100)											
All stages	L, N, A, U	1124	290 (26)	30 (10)		49 (17)	42 (14)	2 (1)		2 (1)		1 (1)			12 (4)	7 (2)		145 (50)

^a^Tick developmental stage: L, larva; N, nymph; A, Adult female; U, undetermined

^b^Ticks were examined for *Borrelia* spp. by the LUX real-time PCR assay

^c^Number of ticks yielding a positive outcome by LUX real-time PCR assay

^d^Abbreviations of *Borrelia* species: *B. a*, *Borrelia afzelii*; *B. g*, *Borrelia garinii*; *B. v*, *Borrelia valaisiana*; *B. b*, *Borrelia burgdorferi* (*s.s*); *B. t*, *Borrelia turdi*; *B. l*, *Borrelia lusitaniae*; *B. m*, *Borrelia miyamotoi*; UT, untypeable

No significant difference was detected between the proportion of *Borrelia* spp.-positive ticks in real-time PCR collected in spring (24.6%, 15 March–15 June) and *Borrelia* spp.-positive ticks collected in autumn (26.5%, 15 July–15 November) (data not shown).

Among the *I. frontalis* ticks, 20.0% (3/15) of the nymphs were real-time PCR positive for *Borrelia* spp. (Table 2), while all larvae were negative (0/8). All 4 *H. marginatum* ticks were real-time PCR negative for *Borrelia* spp., whereas only 1 among 12 *H. punctata* larvae was positive for *Borrelia* spp.

### Prevalence of *Borrelia* species in the ticks

Seven different *Borrelia* species were identified by sequence analysis of the 5S-23S IGS or 16S-23S IGS (Table 2). *B. garinii* was the predominant species and detected in 17% of all *Borrelia*-containing ticks, followed by *B. valaisiana*, 14%; *B. afzelii*, 10%; *B. miyamotoi*, 4.0%; *B. burgdorferi s.s*, 1.0%; *B. lusitaniae*, 1.0% and *B. turdi*, 1.0%, and a mix of different *Borrelia* spp., 2.0%. Based on the sequencing chromatograms, it was not possible to distinguish the specific *Borrelia* spp. involved in the mixed infections. Fifty percent of all *Borrelia* bacteria detected in the ticks were untypeable.

Of all samples that were determined to *Borrelia* species level (*n* = 138; Table 2), 52.9% (*n* = 73) were detected in ticks captured in spring (15 March–15 June), and 47.1% (*n* = 65) were detected in ticks captured in autumn (15 July–15 November) (data not shown). *B. garinii* was more prevalent in the autumn (60.0%, 39/65,) than in spring (20.5%, 15/73 [χ^2^ = 22.47, *df* = 1, *P* < 0.0001]). In contrast, *B. valaisiana* and *B. miyamotoi* were more prevalent in spring than in autumn (*B. valaisiana*: 45.2%, 33/73 vs. 13.8%, 9/65 [χ^2^ = 15.97, *df* = 1, *P* < 0.0001]; *B. miyamotoi.*: 13.7%, 10/73 vs. 3.1%, 2/65 [*P* = 0.03 (OR: 5 CI: 1.216-23.35)]). For the other *Borrelia* species there were no significant differences in seasonal prevalence.

**Fig. 4:**
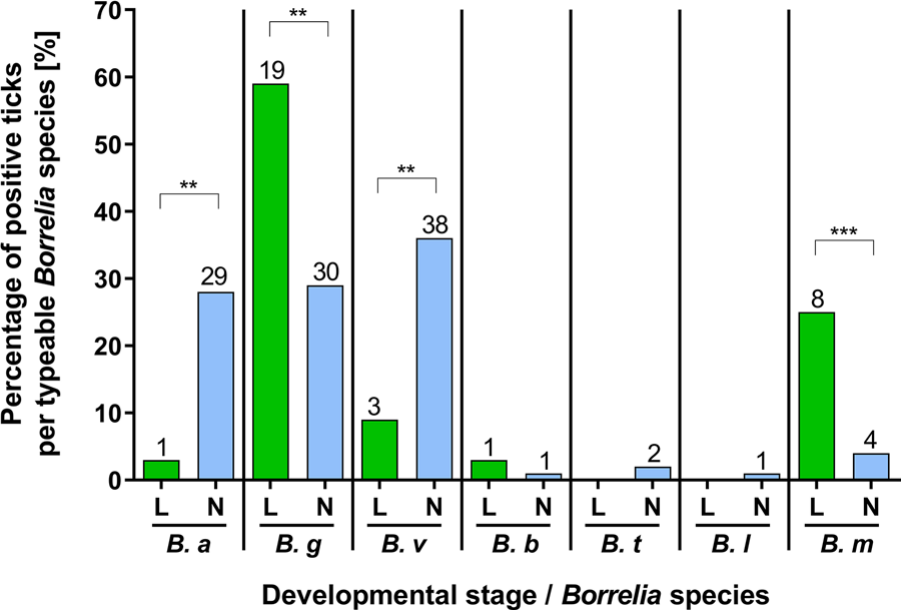
Distribution of *Borrelia* species in larvae (L) and nymphs (N). The percentage of *Borrelia* spp. PCR-positive ticks per typeable species is given. The numbers of infected ticks examined are indicated above bars. **P < 0.01; ***P < 0.001. *B. a*, *B. afzelii*; *B. g*, *B. garinii*; *B. v*, *B. valaisiana*; *B. b*, *B. burgdorferi* (*s.s*); *B. t*, *B. turdi*; *B. l*, *B. lusitaniae*; *B. m*, *B. miyamotoi*

Of all samples that were determined to *Borrelia* species level and where the developmental stage of the corresponding tick could be determined (*n* = 137), 23.4% (*n* = 32) *Borrelia* species were recorded in larvae while 76.6% (*n* = 105) were recorded in nymphs (Table 2). *B. garinii* was more prevalent in larvae (59.4%, 19/32) than in nymphs (28.6%, 30/105 [χ^2^ = 10.13, *df* = 1, *P* = 0.002]) (Fig. 4). *B. miyamotoi* was also more prevalent in larvae (25.0%, 8/32) than in nymphs (3.8%, 4/105 [*P* = 0.001 (OR: 8.417 CI: 2.565-26.25]). *B. afzelii* and *B. valaisiana* showed the opposite pattern and were more prevalent in nymphs (27.6%, 29/105, and 36.2%, 38/105, respectively) than in larvae (3.1%, 1/32 [*P* = 0.003 (OR: 11.89 CI: 1.990-125.2)], and 9.4%, 3/32 [*P* = 0.004 (OR: 5.483 CI: 1.717-17.9)], respectively). There were no significant differences in prevalence between nymphs and larvae among the other *Borrelia* species.

**Fig. 5:**
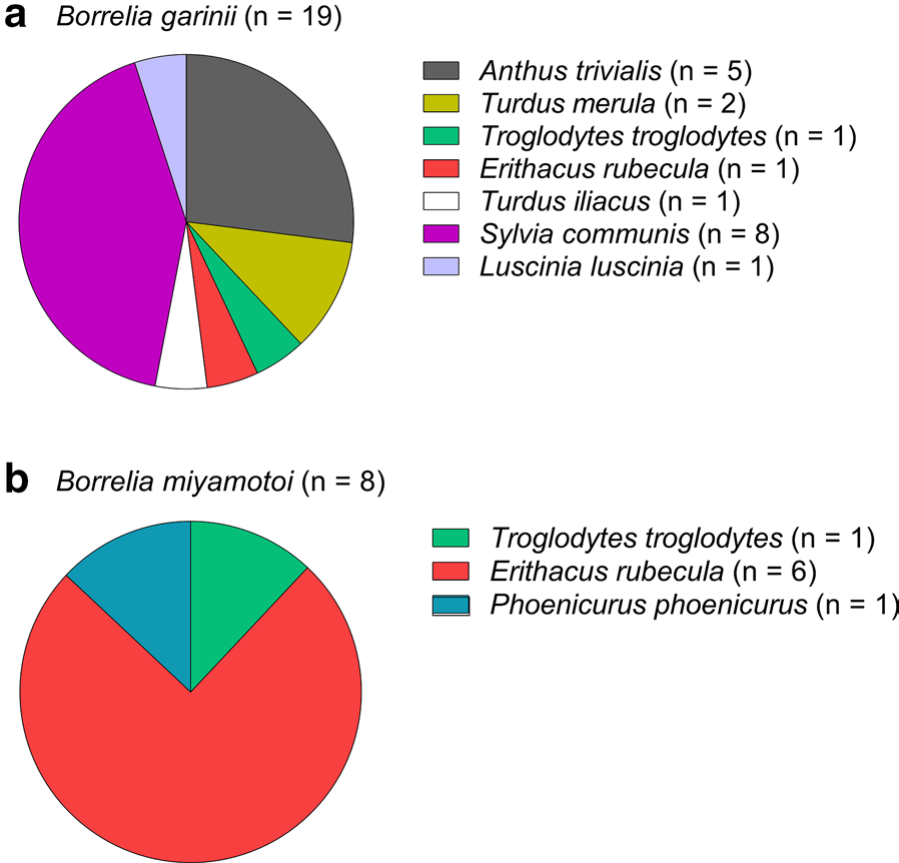
Relative importance of different bird species as carriers of *B. garinii* and *B. miyamotoi* containing larvae. The *Borrelia* species presented were detected in *I. ricinus* larvae removed from birds

High proportions of *I. ricinus* larvae containing *B. garinii* were removed from Common whitethroat (*Sylvia communis*) and *A. trivialis* (Fig. 5a). Also, a high percentage of tick larvae containing *B. miyamotoi* was removed from *E. rubecula* (Fig. 5b).

### Quantity of *Borrelia* cells in the ticks

**Fig. 6:**
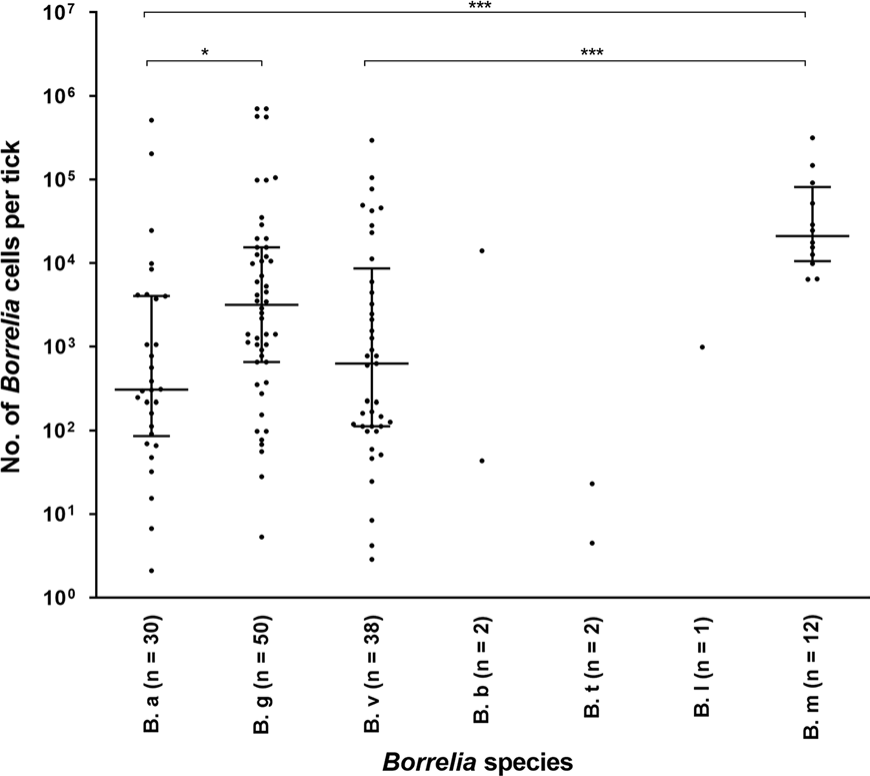
*Borrelia* species plotted against the number of *Borrelia* spp. cells per tick. Horizontal lines indicate the median, with upper and lower quartiles. *P < 0.05; ***P < 0.001. *B. a*, *B. afzelii*; *B. g*, *B. garinii*; *B. v*, *B. valaisiana*; *B. b*, *B. burgdorferi* (*s.s*); *B. t*, *B. turdi*; *B. l*, *B. lusitaniae*; *B. m*, *B. miyamotoi*. Due to few samples, *B. b*, *B. t*, and *B. l* were excluded from the statistical analysis

According to the LUX real-time PCR assay, the number of *Borrelia* cells per tick containing a typeable *Borrelia* species ranged from 2.0 × 10^0^ cells to 7.0 × 10^5^ cells (Fig. 6), with a median of 1.1 × 10^3^. No significant difference in number of *Borrelia* spp. cells between larvae and nymphs was observed (medians 2.5 × 10^3^, and 1.1 × 10^3^, respectively).

Ticks containing *B. miyamotoi* had significantly higher numbers of *Borrelia* cells (median of 2.0 × 10^4^ cells per tick) compared to ticks with *B. afzelii* (median 3.0 × 10^2^, [*Z* = 4.175, *df* = 6, *P* < 0.001]) and compared to ticks with *B. valaisiana* (median 6.0 × 10^2^, [*Z* = 3.824, *df* = 6, *P* < 0.001]) (Fig. 6). Ticks with *B. garinii* had significantly higher numbers of *Borrelia* cells (3.0 × 10^3^ cells per tick) compared to ticks with *B. afzelii* (median 3.0 × 10^2^, [*Z* = 2.644, *df* = 6, *P* = 0.049]). No other significant differences in the number of *Borrelia* cells among *Borrelia* species were detected. Ticks with untypeable *Borrelia*, had a significantly fewer *Borrelia* cells (median of 2.0 × 10^0^) compared to ticks with a typeable *Borrelia* species (median of 1.1 × 10^3^, *P* < 0.0001) (data not shown).

## Discussion

### Tick infestation prevalence on birds

In the present study, tick infestation rates were highest in late April and late September (Fig. 1), when as many as 30% of investigated passerine birds were infested with at least one tick. However, ticks were detected on birds in all study months, with infestation rates in the range of 4.90–31.5%. Observed infestation rates are likely influenced both by the phenology of migration of particular bird species and by tick and host biology. For instance, the early season (March–April) is dominated by short-distance migrants, including important ground foraging tick host species such *T. merula* and *E. rubecula*, while the latter part of spring (May–June) is dominated by long-distance tropical migrants, that to a larger extent comprise insectivorous species that feed less on the ground. In fact, in the present study, most ticks were removed from *E. rubecula*, *T. merula, T. troglodytes*, and *A. trivialis* (Table 1). These results conform to several similar studies, which have revealed that passerine bird species that frequently feed on the ground are more often infested with *I. ricinus* ticks [2–4, 12, 16, 32–35]. In a recent study in Denmark, *E. rubecula, T. merula*, and *P. phoenicurus* constituted less than one third of the birds examined for ticks, but carried 77% of all *I. ricinus* ticks collected; 78% of all 179 *I. ricinus* ticks were collected from birds in the autumn [4]. Similarly, in Switzerland, the prevalence of tick infestation, mainly of *I. ricinus* on migratory birds was significantly higher on birds migrating southwards than northwards [9]. In our study, the highest tick infestation prevalence occurred in April and August–September (Fig. 1).

### Birds as reservoirs and transmission hosts for Borrelia species

A few xenodiagnostic investigations have demonstrated that birds are competent transmission hosts for certain *Borrelia* species. This suggests that birds may harbour infectious *Borrelia* spirochaetes in their skin or blood and consequently can act as competent reservoirs. Another possibility is that birds may be competent transmission hosts in non-spirochaetemic co-feeding transmission among one or more *Borrelia-*containing ticks, feeding adjacent to one or more susceptible ticks. A third possibility is that certain birds may act in both ways of *Borrelia* transmission. Kurtenbach et al. (2002) showed that pheasants are competent transmission hosts for *B. garinii* and *B. valaisiana*, but not for *B. afzelii* [36]. Comstedt et al. (2006) obtained evidence that several species of birds act as transmission hosts for *B. garinii*, thus transmitting the bacteria to larvae of *I. ricinus*, which were ingesting blood from their avian host [2].

The present study may suggest that many *I. ricinus* larvae contracted *B. garinii* and *B. miyamotoi* spirochaetes while feeding on some of their avian hosts (see below). However, while transovarial transmission is rare in *B. burgdorferi* (*s.l.*) [37], it is well known that such transmission is a common trait in *B. miyamotoi*, which results in *B. miyamotoi*-containing larvae capable of transmitting the spirochaetes to their hosts [37]. Thus, it is most likely that *B. miyamotoi*-containing larvae had been transovarially infected whereas the *B. garinii-*containing larvae had contracted the spirochaetes from their avian hosts.

### Tick species

#### Ixodes ricinus

*I. ricinus* is considered to be the primary vector of all human-pathogenic species of *B. burgdorferi* (*s.l.*) and of *B. miyamotoi* occurring in Europe [38]. Moreover, *I. ricinus* is able to transmit *B. turdi* to *T. merula* [32]. Larvae and to a lesser extent nymphs of *I. ricinus* parasitize small mammals, birds, and reptiles; all stages can also be found on medium- and large-sized mammals [39, 40]. In most investigations, the number of larvae of *I. ricinus* collected from birds is usually larger than the number of nymphs collected. However, the proportion of nymphs of *I. ricinus* in relation to that of larvae of *I. ricinus* is usually higher on birds than on small mammals. For instance, during the summer months of 1991–1994 in a study area near Stockholm, Sweden, median numbers of 16–105 larvae and 0–2 nymphs of *I. ricinus* were found on infested bank voles, *Myodes glareolus* [40]. In contrast, from 22,998 birds captured at eight localities in Sweden, Olsen et al. [33] removed 949 *I. ricinus* ticks; 29.8% were larvae and 70.0% were nymphs. Even though the nymphs are easier to detect than the larvae (some of which may therefore have been missed), it is generally accepted that nymphs constitute a significantly higher proportion of the *I. ricinus* ticks on birds than on small mammals. This is confirmed by the results of the present study: almost equal proportions of larvae (48.3%) and nymphs (51.7%) of *I. ricinus* were recorded. Almost equal proportions of larvae and nymphs of *I. ricinus* were also encountered in similar studies by others [4, 41–43]. In the present study 46% of the ticks, most of which were immatures of *I. ricinus*, were removed from *E. rubecula*. Other bird species from which high total numbers of ticks were collected were *T. merula, T. troglodytes*, and *A. trivialis.* The large numbers of ticks from these bird species partly reflects their abundance, where *E. rubecula*, *T. merula*, and *T. troglodytes* (and to a lesser extent *A. trivialis*) make up a large proportion of the trapping totals, but it likely also reflects their habits of spending a lot of time on the ground, thereby rendering them vulnerable to attack by questing larvae and nymphs of *I. ricinus*.

Since the prevalence of tick-associated pathogens is generally higher in tick nymphs than in tick larvae, the high proportion of nymphs feeding on birds suggests that birds receive a substantial proportion of infections from the nymphs. On the other hand, the tick larvae, while feeding on their avian hosts, are likely to contract *Borrelia* bacteria with nymph-derived spirochaetes. In the present study, in *I. ricinus* larvae we detected these *Borrelia* species: *B. afzelii, B. garinii, B. valaisiana, B. burgdorferi* (*s.s.*), and *B. miyamotoi. B. burgdorferi* (*s.l.*) spirochaetes are usually not transmitted transovarially [37]. Therefore, we think it probable that the larvae, containing one or two species in the *B. burgdorferi* s.l. complex (Table 2), had contracted the *Borrelia* cells directly from their infective avian hosts or through co-feeding transmission. In contrast, *B. miyamotoi* is often transmitted transovarially. Therefore, we cannot know if the eight *I. ricinus* larvae positive for *B. miyamotoi* had contracted the spirochaetes transovarially or from their infective avian hosts or by co-feeding transmission.

#### Ixodes frontalis

Among the *Ixodes* ticks that were identified to species level, 2.0% were *I. frontalis* (8 larvae, 15 nymphs, and 1 adult female tick). Untypeable *Borrelia* spp. were detected in 3 among 15 *I. frontalis* nymphs.

*I. frontalis* parasitizes birds, particularly Passeriformes [44], and is a likely enzootic vector of blood-borne microbes in bird-tick-bird cycles. *I. ricinus*, which feeds on both birds and mammals, could here potentially function as a bridge vector by transferring pathogens from the bird-*I. frontalis* enzootic cycle to humans and other mammals. Gilot et al. (1997) describes one occasion when an adult female of *I. frontalis* attached to a human tick-collector’s hand [45]. This is apparently an exceptionally rare behaviour exhibited by this otherwise strictly ornithophagous tick species. In western and central France and in north-eastern Spain, *I. frontalis* is the most abundant ixodid on passerine birds [46, 47]. Heylen et al. (2016) have shown that *I. frontalis* is able to transmit *B. turdi* to *T. merula*, but that *I. frontalis* appears to be an incompetent vector of *B. garinii* and *B. valaisiana* [32].

Agoulon and co-workers showed that the seasonal activity of *I. frontalis* differs from that of *I. ricinus*, particularly of the larvae. In *I. frontalis* larval activity in France was completely absent during the summer [47]. This conforms to our results: immatures of *I. ricinus* were recorded during both spring–early summer and late summer–autumn while *I. frontalis* was only present during the first of these seasons.

#### Haemaphysalis punctata

The adults of *Haemaphysalis punctata* are usually parasites of medium and large mammals, in particular domestic ungulates, but may occasionally feed on humans [39, 44, 48, 49]. The immatures can be found on small mammals and more rarely on birds and lizards [39, 44, 48]. In the present study, we recorded 12 larvae of *H. punctata*, 11 of which were investigated and scored negative for *Borrelia* spp. (Table 1). Previously, among 967 ticks removed from 465 tick-infested birds out of nearly 23,000 birds examined, only 1 *H. punctata*, a nymph, was found [2]. An earlier study on questing ticks at three Swedish Baltic Sea islands, spirochaetes, believed to belong to *B. burgdorferi* (*s.l.*) were recorded in ~ 2% of *H. punctata* nymphs [50]. Since the spirochaetes were detected by phase-contrast microscopy, it is not certain that they belong to *B. burgdorferi* (*s.l.*). In any case, the low number of spirochaetes detected in the ticks and the low prevalence suggest that *H. punctata* is not an important *Borrelia* spp. vector [50]. This conclusion conforms to our results.

#### Hyalomma marginatum

Each spring *Hyalomma marginatum* is introduced to northern Europe when larvae or nymphs are carried by birds migrating from southern Europe to Sweden and neighbouring countries [39, 51]. This is reflected in our data by the fact that *H. marginatum* was only present on birds captured during April and May (Fig. 2b). During the summer of 2018, which was exceptionally warm, there were several records of adult *H. marginatum* and *H. rufipes* in Sweden [52]. These adult ticks are believed to originally have infested avian hosts in the Mediterranean region, then feeding and becoming nymphs while still on their hosts flying northwards for several days. Subsequently, the blood-fed nymphs left their hosts after their arrival in southern or central Sweden. Due to the warm summer of 2018 such *H. marginatum* and *H. rufipes* nymphs in Sweden were able to reach the adult stage before the end of the summer. However, these tick species are presumably not yet permanent members of the Swedish tick fauna. *H. marginatum* and *H. rufipes* are of great importance as primary vectors of CCHFV [53, 54]. In the present study, one larva, two nymphs, and one adult female of *H. marginatum* were recorded. All were negative for *Borrelia*. It is noteworthy that not only larvae but even nymphs and adult females can be transported into Sweden by birds. The fact that one adult female tick was encountered on a *Fringilla coelebs* suggests that, if/when the climate in southern Sweden becomes optimal for *H. marginatum* and *H. rufipes,* they will presumably establish indigenous populations here.

### *Borrelia garinii* and *Borrelia valaisiana*

Birds are reservoirs and transmission hosts for human-pathogenic *B. garinii* and *B. valaisiana* [5, 11, 38, 55]. As shown in Table 2, most *Borrelia-*containing *Ixodes* larvae were positive for *B. garinii*. We believe that these larvae had contracted the *B. garinii* bacteria from their avian hosts. However, in a few cases where one or more *Borrelia*-containing nymphs were feeding together with larvae on the same bird, it cannot be excluded that the larvae contracted the spirochaetes by co-feeding transmission. The majority (69%) of the *I. ricinus* larvae with *B. garinii* were removed from either *S. communis* (42%) or *A. trivialis* (27%), which may indicate that these bird species may be competent reservoirs and/or transmission hosts for *B. garinii*. Among the species in the *B. burgdorferi* (*s.l.*) complex present in Europe, *B. garinii* has the greatest potential to cause severe late neurological disease manifestations. Therefore, from a public health point of view it can be considered to be the most important *Borrelia* species in Europe. This also underlines the view that birds have a considerable, although indirect, impact on the epidemiology of tick-borne human diseases. *B. valaisiana* has been found to be common in ticks removed from birds in Europe [2]. Surprisingly, only a few (*n* = 3) *Borrelia*-containing *I. ricinus* larvae removed from the birds in the present study proved to contain *B. valaisiana* spirochaetes. However, it should be noted that 46 larvae (58%) contained untypeable *Borrelia* bacteria. It is possible that some of them were due to *B. valaisiana.*

#### Borrelia afzelii

*Borrelia afzelii* was present in several nymphs of *I. ricinus*, but only in one larva. *B. afzelii* is generally considered to use small mammals as its vertebrate reservoir [38]. However, there are many reports describing the presence of *B. afzelii* in *I. ricinus* larvae removed from birds [38, 56, 57]. The reason(s) for the presence of *B. afzelii* in tick larvae attached to birds need(s) further investigations. The presence of *B. afzelii* in *Ixodes* nymphs removed from birds is most likely because these ticks, in their larval stage, had fed on *B. afzelii*-infected small mammals.

#### Borrelia lusitaniae

There are a few cases of human Lyme borreliosis where *B. lusitaniae* is considered the etiological agent. It has lizards of the Lacertidae family as its vertebrate reservoir and *I. ricinus* as its vector [58, 59]. *B. lusitaniae* is considered to have originated in Portugal from where it has spread to other countries in southern Europe and to North Africa and to scattered localities in Central, Eastern and South-Eastern Europe. DNA of this reptile-associated *Borrelia* species has even been demonstrated on a few occasions in adults and nymphs of *I. ricinus* that had fed on humans in southern and south-central Sweden [27]. The main method by which this *Borrelia* species has spread is thought to be by birds infested with *B. lusitaniae*-containing ticks. It has been recorded in Switzerland from *I. ricinus* larvae and nymphs feeding on *E. rubecula* and from larvae feeding on *Turdus philomelos, T. merula* and *Phoenicurus phoenicurus* [9].

#### Borrelia turdi

Birds are considered as reservoirs and transmission hosts for *B. turdi.* In Europe, *I. frontalis* is considered to be the primary vector of *B. turdi* [11, 32]. We did not record any among 16 *I. frontalis* specimens positive for *B. turdi*. However, 2 among 549 nymphs of *I. ricinus* were positive for this species*.* To our knowledge, *B. turdi* has not previously been recorded from ticks or birds captured in Sweden. However, *B. turdi* was first recorded in Europe by Hasle et al. (2011) in Norway [3], where they detected *B. turdi* in 0.4% of *I. ricinus* larvae and 0.4% of *I. ricinus* nymphs collected from northward-migrating passerine birds. *B. turdi* was then detected in *I. frontalis, I. ricinus* and *H. punctata* removed from birds in northern Spain [60], from *I. ricinus* removed from *T. merula* in Poland [5], from *I. frontalis* removed from *T. merula* captured in the Archipelago of the Azores [35], from *I. frontalis* feeding on birds in Belgium [61], and from *I. frontalis* feeding on *T. merula, T. philomelos, P. major*, and *T. troglodytes* in Portugal [62]. Prevalences of 7% to 56% of *B. turdi* in *I. frontalis* have been recorded [62]. *B. turdi* has also been detected in skin biopsies from *T. merula,* which is a competent transmission host for this bacterium [11, 62]. There is not yet any indication that *B. turdi* infects humans.

#### *Borrelia burgdorferi* (*s.s.*)

Only one *I. ricinus* larva and one *I. ricinus* nymph were positive for *B. burgdorferi* (*s.s.*). Several studies have shown that birds are competent reservoirs and transmission hosts for North American strains of *B. burgdorferi* (*s.s.*). [38]. *B. burgdorferi* (*s.s.*) is responsible for the majority of cases of Lyme borreliosis in North America. In Europe, *B. burgdorferi* (*s.s.*) is recorded rarely in ticks removed from birds [2, 5, 38, 57]. However, as pointed out by Franke et al. [56], this may just reflect that this species is generally rare in many European regions, rather than suggesting that birds are incompetent reservoirs for European strains of *B. burgdorferi* (*s.s.*).

#### Borrelia miyamotoi

*Borrelia miyamotoi* is transmitted transovarially from the adult female tick to her offspring, which results in *B. miyamotoi*-containing tick larvae [37]. Transovarial transmission is much rarer or absent in the species within the *B. burgdorferi* (*s.l.*) complex [37]. In the present study, eight *I. ricinus* larvae were positive for *B. miyamotoi.* Presence of spirochaetes in tick larvae infesting birds can occur if the avian host is infected with spirochaetes, if co-feeding transmission from a *Borrelia*-containing tick to an adjacent susceptible tick larva takes place or if transovarial transmission of spirochaetes from an adult female tick to her offspring occurs. Detection of spirochaetes in unfed *Ixodes* larvae is an indication of transovarial transmission, which is a prevalent trait in *B. miyamotoi* [37, 63]. This species is known to have rodents as one of its vertebrate reservoirs, but birds are considered to be another of its reservoirs [63, 64].

It is reasonable to assume that the infectiousness of a bacterial pathogen is, in general, positively correlated to the concentration of cells of the bacterium in the infective medium. Our results revealed a significantly higher median density of cells in *B. miyamotoi-*containing ticks than in *B. burgdorferi* (*s.l.*)-containing ticks. In another study, based on ticks infesting humans, Wilhelmsson et al. also recorded a higher median density of spirochaetes in *B. miyamotoi*-containing ticks than in *B. burgdorferi* (*s.l.*)-containing ticks [27]. We conjecture that this may be an evolutionary trait which compensates for the generally lower prevalence of *B. miyamotoi* in the tick population compared to that of *B. burgdorferi* (*s.l.*). Similarly, it has previously been shown that in rodents infected with either *B. miyamotoi* or *B. burgdorferi*, the concentration of cells in the blood was generally much higher in the *B. miyamotoi-*infected rodents [63]. It is important to realise that, since transovarial transmission is a common trait in *B. miyamotoi,* which results in *B. miyamotoi*-containing tick larvae, many of which will infest birds, this *Borrelia* can be dispersed to distant localities.

### Tick-borne encephalitis virus in ticks infesting birds

Proven, competent vertebrate transmission hosts of TBEV are many species of small mammals (rodents, insectivores) while, in view of recorded viremia and/or virus isolations, some ungulates (goat, sheep) and many bird species [65, 66] may be considered suspected or presumed transmission-competent hosts for this virus. Moreover, there are a number of records of TBEV from ticks infesting birds [20, 34, 66, 67]. The prevalence of this virus in host-seeking *I. ricinus* and in ticks removed from hosts in TBEV-enzootic regions is usually < 1% [68]. The absence of TBEV in all bird-derived ticks investigated by us thus conforms to similar studies, which have recorded a low or zero prevalence of this virus.

## Conclusions

The results corroborate the view that the contribution of birds to human Lyme borreliosis is substantial. Several ground-foraging bird species are directly and indirectly important for the local maintenance and long-distance dispersal of human-pathogenic or potentially pathogenic *Borrelia* bacteria. Our data suggest that *S. communis* and *A. trivialis* are reservoirs and/or transmission hosts for *B. garinii.* Many ground-foraging bird species are important tick hosts, which significantly contribute to the local maintenance of tick populations and to long-distance dispersal of several tick species.

## Supplementary information

**Additional file 1: Fig. S1.** The aligned *Borrelia* nucleotide sequences based on PCR products.

## Data Availability

The data supporting the conclusions of this article are included within the article. Raw data can be shared with researchers upon a specific request.
