# Supplementary material for: Migratory birds as disseminators of ticks and the tick-borne pathogens Borrelia bacteria and tick-borne encephalitis (TBE) virus: a seasonal study at Ottenby Bird Observatory in South-eastern Sweden
Source: Parasit Vectors. 2020 Dec 3;13:607. doi: 10.1186/s13071-020-04493-5 (PMC7713317; doi:10.1186/s13071-020-04493-5)
Supplement: Supplementary file 1 — Additional file 1: Fig. S1. The aligned Borrelia nucleotide sequences based on PCR products. [file 13071_2020_4493_MOESM1_ESM.docx]

**Additional file 1. Aligned *Borrelia* nucleotide sequences based on PCR-products**

The identifier line, which begins with '>', gives the name of the **sample ID_microorganism_gene target**

**>15_24_Borrelia miyamotoi_16S-23S ribosomal RNA intergenic spacer, partial sequence**

CTAAGAAATTGTTTAAATTAATTATTATTTTAACTCTTCCCTTGTTTTCTCAAAGGCTTTTTTGGGGGTTTAGCTCAGTTGGTTTAGAGCATCGGCTTTGCAAGCCGGGGGTCAAGGGTTCGAGTCCCTTAACCTCCATTTATTTATTGTTAAATAGGTATGCAGCCTTTTAAGAAATGGCTTTGTTTTATATTGAGTGAATGAATTTTTCATGGCTTTAAAAGATGTGTTTTTATGTTGTTTGTTGTCTTGATGAATTGCTTTTTATGAATTCCAAAACTTGATTACATGCAAAAAGTTTATCTCTAATTATTTTATTAAGGTTATTAAGGACCTGATCATGAATCAACCTAATTGGATTGGGATCAGGAGGAGTTGAACC

**>16_19_Borrelia miyamotoi_16S-23S ribosomal RNA intergenic spacer, partial sequence**

CTAAGAGAAAGAAATTGTTTAAATTAATTATTATTTTAACTCTTCCCTTGTTTTCTCAAAGGCTTTTTTGGGGGTTTAGCTCAGTTGGTTTAGAGCATCGGCTTTGCAAGCCGGGGGTCAAGGGTTCGAGTCCCTTAACCTCCATTTATTTATTGTTAAATAGGTATGCAGCCTTTTAAGAAATGGCTTTGTTTTATATTGAGTGAATGAATTTTTCATGGCTTTAAAAGATGTGTTTTTATGTTGTTTGTTGTCTTGATGAATTGCTTTTTATGAATTCCAAAACTTGATTACATGCAAAAAGTTTATCTCTAATTATTTTATTAAGGTTATTAAGGACCTGATCATGAATCAACCTAATTGGATTGGGATCAGGAGGAGTTGAACC

**>22_20_Borrelia miyamotoi_16S-23S ribosomal RNA intergenic spacer, partial sequence**

CTAAGAGAAAGAAATTGTTTAAATTAATTATTATTTTAACTCTTCCCTTGTTTTCTCAAAGGCTTTTTTGGGGGTTTAGCTCAGTTGGTTTAGAGCATCGGCTTTGCAAGCCGGGGGTCAAGGGTTCGAGTCCCTTAACCTCCATTTATTTATTGTTAAATAGGTATGCAGCCTTTTAAGAAATGGCTTTGTTTTATATTGAGTGAATGAATTTTTCATGGCTTTAAAAGATGTGTTTTTATGTTGTTTGTTGTCTTGATGAATTGCTTTTTATGAATTCCAAAACTTGATTACATGCAAAAAGTTTATCTCTAATTATTTTATTAAGGTTATTAAGGACCTGATCATGAATCAACCTAATTGGATTGGGATCAGGAGGAGTTGAACC

**>24_6_Borrelia miyamotoi_16S-23S ribosomal RNA intergenic spacer, partial sequence**

CTAAGAGAAAGAAATTGTTTAAATTAATTATTATTTTAACTCTTCCCTTGTTTTCTCAAAGGCTTTTTTGGGGGTTTAGCTCAGTTGGTTTAGAGCATCGGCTTTGCAAGCCGGGGGTCAAGGGTTCGAGTCCCTTAACCTCCATTTATTTATTGTTAAATAGGTATGCAGCCTTTTAAGAAATGGCTTTTTTTTATATTGAGTGAATGAATTTTTCATGGCTTTAAAAGATGTGTTTTTATGTTGTTTGTTGTCTTGATGAATTGCTTTTTATGAATTCCAAAACTTGATTACATGCAAAAAGTTTATCTCTAATTATTTTATTAAGGTTATTAAGGACCTGATCATGAATCAACCTAATTGGATTGGGATCAGGAGGAGTTGAACC

**>32_19_Borrelia miyamotoi_16S-23S ribosomal RNA intergenic spacer, partial sequence**

CTAAGAGAAAGAAATTGTTTAAATTAATTATTATTTTAACTCTTCCCTTGTTTTCTCAAAGGCTTTTTTGGGGGTTTAGCTCAGTTGGTTTAGAGCATCGGCTTTGCAAGCCGGGGGTCAAGGGTTCGAGTCCCTTAACCTCCATTTATTTATTGTTAAATAGGTATGCAGCCTTTTAAGAAATGGCTTTGTTTTATATTGAGTGAATGAATTTTTCATGGCTTTAAAAGATGTGTTTTTATGTTGTTTGTTGTCTTGATGAATTGCTTTTTATGAATTCCAAAACTTGATTACATGCAAAAAGTTTATCTCTAATTATTTTATTAAGGTTATTAAGGACCTGATCATGAATCAACCTAATTGGATTGGGATCAGGAGGAGTTGAACC

**>33_19_Borrelia miyamotoi_16S-23S ribosomal RNA intergenic spacer, partial sequence**

CTAAGAGAAAGAAATTGTTTAAATTAATTATTATTTTAACTCTTCCCTTGTTTTCTCAAAGGCTTTTTTGGGGGTTTAGCTCAGTTGGTTTAGAGCATCGGCTTTGCAAGCCGGGGGTCAAGGGTTCGAGTCCCTTAACCTCCATTTATTTATTGTTAAATAGGTATGCAGCCTTTTAAGAAATGGCTTTGTTTTATATTGAGTGAATGAATTTTTCATGGCTTTAAAAGATGTGTTTTTATGTTGTTTGTTGTCTTGATGAATTGCTTTTTATGAATTCCAAAACTTGATTACATGCAAAAAGTTTATCTCTAATTATTTTATTAAGGTTATTAAGGACCTGATCATGAATCAACCTAATTGGATTGGGATCAGGAGGAGTTGAACC

**>36_8_Borrelia miyamotoi_16S-23S ribosomal RNA intergenic spacer, partial sequence**

CTAAGAGAAAGAAATTGTTTAAATTAATTATTATTTTAACTCTTCCCTTGTTTTCTCAAAGGCTTTTTTGGGGGTTTAGCTCAGTTGGTTTAGAGCATCGGCTTTGCAAGCCGGGGGTCAAGGGTTCGAGTCCCTTAACCTCCATTTATTTATTGTTAAATAGGTATGCAGCCTTTTAAGAAATGGCTTTGTTTTATATTGAGTGAATGAATTTTTCATGGCTTTAAAAGATGTGTTTTTATGTTGTTTGTTGTCTTGATGAATTGCTTTTTATGAATTCCAAAACTTGATTACATGCAAAAAGTTTATCTCTAATTATTTTATTAAGGTTATTAAGGACCTGATCATGAATCAACCTAATTGGATTGGGATCAGGAGGAGTTGAACC

**>36_20_Borrelia miyamotoi_16S-23S ribosomal RNA intergenic spacer, partial sequence**

CTAAGAGAAAGAAATTGTTTAAATTAATTATTATTTTAACTCTTCCCTTGTTTTCTCAAAGGCTTTTTTGGGGGTTTAGCTCAGTTGGTTTAGAGCATCGGCTTTGCAAGCCGGGGGTCAAGGGTTCGAGTCCCTTAACCTCCATTTATTTATTGTTAAATAGGTATGCAGCCTTTTAAGAAATGGCTTTGTTTTATATTGAGTGAATGAATTTTTCATGGCTTTAAAAGATGTGTTTTTATGTTGTTTGTTGTCTTGATGAATTGCTTTTTATGAATTCCAAAACTTGATTACATGCAAAAAGTTTATCTCTAATTATTTTATTAAGGTTATTAAGGACCTGATCATGAATCAACCTAATTGGATTGGGATCAGGAGGAGTTGAACC

**>38_21_Borrelia miyamotoi_16S-23S ribosomal RNA intergenic spacer, partial sequence**

CTAAGAGAAAGAAATTGTTTAAATTAATTATTATTTTAACTCTTCCCTTGTTTTCTCAAAGGCTTTTTTGGGGGTTTAGCTCAGTTGGTTTAGAGCATCGGCTTTGCAAGCCGGGGGTCAAGGGTTCGAGTCCCTTAACCTCCATTTATTTATTGTTAAATAGGTATGCAGCCTTTTAAGAAATGGCTTTGTTTTATATTGAGTGAATGAATTTTTCATGGCTTTAAAAGATGTGTTTTTATGTTGTTTGTTGTCTTGATGAATTGCTTTTTATGAATTCCAAAACTTGATTACATGCAAAAAGTTTATCTCTAATTATTTTATTAAGGTTATTAAGGACCTGATCATGAATCAACCTAATTGGATTGGGATCAGGAGGAGTTGAACC

**>40_18_Borrelia miyamotoi_16S-23S ribosomal RNA intergenic spacer, partial sequence**

CTAAGAGAAAGAAATTGTTTAAATTAATTATTATTTTAACTCTTCCCTTGTTTTCTCAAAGGCTTTTTTGGGGGTTTAGCTCAGTTGGTTTAGAGCATCGGCTTTGCAAGCCGGGGGTCAAGGGTTCGAGTCCCTTAACCTCCATTTATTTATTGTTAAATAGGTATGCAGCCTTTTAAGAAATGGCTTTGTTTTATATTGAGTGAATGAATTTTTCATGGCTTTAAAAGATGTGTTTTTATGTTGTTTGTTGTCTTGATGAATTGCTTTTTATGAATTCCAAAACTTGATTACATGCAAAAAGTTTATCTCTAATTATTTTATTAAGGTTATTAAGGACCTGATCATGAATCAACCTAATTGGATTGGGATCAGGAGGAGTTGAACC

**>44_5_Borrelia miyamotoi_16S-23S ribosomal RNA intergenic spacer, partial sequence**

CTAAGAGAAAGAAATTGTTTAAATTAATTATTATTTTAACTCTTCCCTTGTTTTCTCAAAGGCTTTTTTGGGGGTTTAGCTCAGTTGGTTTAGAGCATCGGCTTTGCAAGCCGGGGGTCAAGGGTTCGAGTCCCTTAACCTCCATTTATTTATTGTTAAATAGGTATGCAGCCTTTTAAGAAATGGCTTTGTTTTATATTGAGTGAATGAATTTTTCATGGCTTTAAAAGATGTGTTTTTATGTTGTTTGTTGTCTTGATGAATTGCTTTTTATGAATTCCAAAACTTGATTACATGCAAAAAGTTTATCTCTAATTATTTTATTAAGGTTATTAAGGACCTGATCATGAATCAACCTAATTGGATTGGGATCAGGAGGAGTTGAACC

**>45_18_Borrelia miyamotoi_16S-23S ribosomal RNA intergenic spacer, partial sequence**

CTAAGAGAAAGAAATTGTTTAAATTAATTATTATTTTAACTCTTCCCTTGTTTTCTCAAAGGCTTTTTTGGGGGTTTAGCTCAGTTGGTTTAGAGCATCGGCTTTGCAAGCCGGGGGTCAAGGGTTCGAGTCCCTTAACCTCCATTTATTTATTGTTAAATAGGTATGCAGCCTTTTAAGAAATGGCTTTGTTTTATATTGAGTGAATGAATTTTTCATGGCTTTAAAAGATGTGTTTTTATGTTGTTTGTTGTCTTGATGAATTGCTTTTTATGAATTCCAAAACTTGATTACATGCAAAAAGTTTATCTCTAATTATTTTATTAAGGTTATTAAGGACCTGATCATGAATCAACCTAATTGGATTGGGATCAGGAGGAGTTGAACC

**>13_1_Borrelia burgdorferi sensu stricto_5S-23S ribosomal RNA intergenic spacer, partial sequence**

GAGTTCGCGGGAGAGTAGGTTATTGCCAGGGTTTTTATTTTTATACTTTAAACTTTGATTTTATTTTTATGTTTTTTAAATATTGGTGTTTTTGAATGTGTTGTTTAAATAACATAAAAAATAAAATATATATTGACATGTATTAAACAAAGATATATATTATTTTATGTTGTATAAATAAATTGGCAAAATAGAGATGGAAGATAAAAATATGGTCAAAGTAATAAGAGTCTATGGTGAATGCC

**>30_22_Borrelia burgdorferi sensu stricto_5S-23S ribosomal RNA intergenic spacer, partial sequence**

GAGTTCGCGGGAGAGTAGGTTATTGCCAGGGTTTTTATTTTTATACTTTAAACTTTGATTTTATTTTTAAGTTTTTTAAATATTGGTGTTTTTGAATGTGTTGTTTAAATAACATAAAAAATAAAATATATATATTGACATGCATTAAACAAAGATATATATTATTTTATGTTGTATAAATAAATTGGCAAAATAGAGATGGAAGATAAAAATATGGTCAAAGTAATAAGAGTCTATGGTGAATGCC

**>20_4_Borrelia lusitaniae_5S-23S ribosomal RNA intergenic spacer, partial sequence**

GAGTTCGCGGGAGAGTAGGTTATTGCCAGGGTTTTTTATTTTATACTTCAAATCTTGAATTTATTTTTTATGTTTTTTAAATGTTCATTTTTTTGAATGTTTGATTTAAAAAAATATAAAAAATAAAATAGATATTGACATGGATTAAACAAAGATATATATTATTCTATGTTGCATAAACAAATTGGCAAAGTAGAGATGGAAGATAAAAATATGGTCAAAGTAATAAGAGTCTATGGTGAATGCC

**>7_1_Borrelia turdi_5S-23S ribosomal RNA intergenic spacer, partial sequence**

GAGTTCGCGGGAGAGTAAGTTATTGCCAGGGTTTTTATTTTATACTTTAAGCTCTTAATTTATTTTTTATGTTTTTTAAATATTCAAAAAAATGAATATTTAAAAAACATAAAAAATAAATTAGATATTGACATAGATTAAACAAAAATATATATTATTCTATGTTGCATAAACAAATTGGCAAAGTAGAGACGGAAGATAAAAATATGGTCAAAGTAATAAGAGTCTATGGTGAATGCC

**>37_1_Borrelia turdi_5S-23S ribosomal RNA intergenic spacer, partial sequence**

GAGTTCGCGGGAGAGTAAGTTATTGCCAGGGTTTTTATTTTATACTTTAAGCTCTTAATTTATTTTTTATGTTTTTAAATATTCATTTTTTTGAATATTTAAAAACATAAAAAATAAATTAGATATTGACATAGATTAAACAAAAATATATATTATTCTATGTTGCATAAACAAATTGGCAAAGTAGAGATGGAAGATAAAAATATGGTCAAAGTAATAAGAGTCTATGGTGAATGCC

**>6_1_Borrelia valaisiana_5S-23S ribosomal RNA intergenic spacer, partial sequence**

GAGTTCGCGGGAGAGTAAGTTATTGCCAGGGTTTTTATTTTGTAATTTAAACCTTAAATTTATTTTTTATATTTTTTTAATGTTCATGTTTTTGAATGTTTTATTCAAATAATGTAAAAAATAAAATAGATATTGACATGGATTGAACAAAAGATATATATTATTTTATGTTGCATAAACAAATTGGCAAAATAGAGATGGAAGATAAAAATATGGTCAAAGTAATAAGAGTCTATGGTGAATGCC

**>9_1_Borrelia valaisiana_5S-23S ribosomal RNA intergenic spacer, partial sequence**

GAGTTCGCGGGAGAGTAAGTTATTGCCAGGGTTTTTATTTTGTAATTTAAACCTTAAATTTATTTTTTATATTTTTTTAATGTTCATGTTTTTGAATGTTTTATTCAAATAATGTAAAAAATAAAATAGATATTGACATGGATTGAACAAAAGATATATATTATTTTATGTTGCATAAACAAATTGGCAAAATAGAGATGGAAGATAAAAATATGGTCAAAGTAATAAGAGTCTATGGTGAATGCC

**>12_1_Borrelia valaisiana_5S-23S ribosomal RNA intergenic spacer, partial sequence**

GAGTTCGCGGGAGAGTAAGTTATTGCCAGGGTTTTTATTTTGTAATTTAAACCTTAAATTTATTTTTTATATTTTTTTAATGTTTATGTTTTTGAATGTTTTATTCAAATAATGTAAAAAATAAAATAGATATTGACATGGATTGAACAAAAGATATATATTATTTTATGTTGCATAAACAAATTGGCAAAATAGAGATGGAAGATAAAAATATGGTCAAAGTAATAAGAGTCTATGGTGAATGCC

**>14_1_Borrelia valaisiana_5S-23S ribosomal RNA intergenic spacer, partial sequence**

GAGTTCGCGGGAGAGTAAGTTATTGCCAGGGTTTTTATTTTGTAATTTAAACCTTAAATTTATTTTTTATATTTTTTTAATGTTCATGTTTTTGAATGTTTTATTCAAATAATGTAAAAAATAAAATAGATATTGACATGGATTGAACAAAAGATATATATTATTTTATGTTGCATAAACAAATTGGCAAAATAGAGATGGAAGATAAAAATATGGTCAAAGTAATAAGAGTCTATGGTGAATGCC

**>18_1_Borrelia valaisiana_5S-23S ribosomal RNA intergenic spacer, partial sequence**

GAGTTCGCGGGAGAGTAAGTTATTGCCAGGGTTTTTATTTTGTAATTTAAACCTTAAATTTATTTTTTATATTTTTTTAATGTTCATGTTTTTGAATGTTTTATTCAAATAATGTAAAAAATAAAATAGATATTGACATGGATTGAACAAAAGATATATATTATTTTATGTTGCATAAACAAATTGGCAAAATAGAGATGGAAGATAAAAATATGGTCAAAGTAATAAGAGTCTATGGTGAATGCC

**>19_1_Borrelia valaisiana_5S-23S ribosomal RNA intergenic spacer, partial sequence**

GAGTTCGCGGGAGAGTAAGTTATTGCCAGGGTTTTTATTTTGTACTTTAAACCTTAAATTTATTTTTTATATTTTTTTAATGTTCATGTTTTTGAATGTTTTATTCAAATAATGTAAAAAATAAAATAGATATTGACATGGATTGAACAAAAGATATATATTATTTTATGTTGCATAAACAAATTGGCAAAATAGAGATGGAAGATAAAAATATGGTCAAAGTAATAAGAGTCTATGGTGAATGCC

**>20_1_Borrelia valaisiana_5S-23S ribosomal RNA intergenic spacer, partial sequence**

GAGTTCGCGGGAGAGTAAGTTATTGCCAGGGTTTTTATTTTGTAATTTAAACCTTAAATTTATTTTTTATATTTTTTTAATGTTCATGTTTTTGAATGTTTTATTCAAATAATGTAAAAAATAAAATAGATATTGACATGGATTGAACAAAAGATATATATTATTTTATGTTGCATAAACAAATTGGCAAAATAGAGATGGAAGATAAAAATATGGTCAAAGTAATAAGAGTCTATGGTGAATGCC

**>35_1_Borrelia valaisiana_5S-23S ribosomal RNA intergenic spacer, partial sequence**

GAGTTCGCGGGAGAGTAAGTTATTGCCAGGGTTTTTATTTTGTACTTTAAACCTTAAATTTATTTTTTATATTTTTTTAATGTTCATGTTTTTGAATGTTTTATTCAAATAATGTAAAAAATAAAATAGATATTGACATGGATTGAACAAAAGATATATATTATTTTATGTTGCATAAACAAATTGGCAAAATAGAGATGGAAGATAAAAATATGGTCAAAGTAATAAGAGTCTATGGTGAATGCC

**>38_1_Borrelia valaisiana_5S-23S ribosomal RNA intergenic spacer, partial sequence**

GAGTTCGCGGGAGAGTAAGTTATTGCCAGGGTTTTTATTTTGTAATTTAAACCTTAAATTTATTTTTTATATTTTTTTAATGTTTATGTTTTTGAATGTTTTATTCAAATAATGTAAAAAATAAAATAGATATTGACATGGATTGAACAAAAGATATATATTATTTTATGTTGCATAAACAAATTGGCAAAATAGAGATGGAAGATAAAAATATGGTCAAAGTAATAAGAGTCTATGGTGAATGCC

**>42_1_Borrelia valaisiana_5S-23S ribosomal RNA intergenic spacer, partial sequence**

GAGTTCGCGGGAGAGTAAGTTATTGCCAGGGTTTTTATTTTGTAATTTAAACCTTAAATTTATTTTTTATATTTTTTTAATGTTCATGTTTTTGAATGTTTTATTCAAATAATGTAAAAAATAAAATAGATATTGACATGGATTGAACAAAAGATATATATTATTTTATGTTGCATAAACAAATTGGCAAAATAGAGATGGAAGATAAAAATATGGTCAAAGTAATAAGAGTCTATGGTGAATGCC

**>45_1_Borrelia valaisiana_5S-23S ribosomal RNA intergenic spacer, partial sequence**

GAGTTCGCGGGAGAGTAAGTTATTGCCAGGGTTTTTATTTTGTACTTTAAACCTTAAATTTATTTTTTATATTTTTTTAATGTTCATGTTTTTGAATGTTTTATTCAAATAATGTAAAAAATAAAATAGATATTGACATGGATTGAACAAAAGATATATATTATTTTATGTTGCATAAACAAATTGGCAAAATAGAGATGGAAGATAAAAATATGGTCAAAGTAATAAGAGTCTATGGTGAATGCC

**>46_1_Borrelia valaisiana_5S-23S ribosomal RNA intergenic spacer, partial sequence**

GAGTTCGCGGGAGAGTAAGTTATTGCCAGGGTTTTTATTTTGTAATTTAAACCTTAAATTTATTTTTTATATTTTTTTAATGTTCATGTTTTTGAATGTTTTATTCAAATAATGTAAAAAATAAAATAGATATTGACATGGATTGAACAAAAGATATATATTATTTTATGTTGCATAAACAAATTGGCAAAATAGAGATGGAAGATAAAAATATGGTCAAAGTAATAAGAGTCTATGGTGAATGCC

**>1_2_Borrelia valaisiana_5S-23S ribosomal RNA intergenic spacer, partial sequence**

GAGTTCGCGGGAGAGTAAGTTATTGCCAGGGTTTTTATTTTGTAATTTAAACCTTAAATTTATTTTTTATATTTTTTTAATGTTCATGTTTTTGAATGTTTTATTCAAATAATGTAAAAAATAAAATAGATATTGACATGGATTGAACAAAAGATATATATTATTTTATGTTGCATAAACAAATTGGCAAAATAGAGATGGAAGATAAAAATATGGTCAAAGTAATAAGAGTCTATGGTGAATGCC

**>6_2_Borrelia valaisiana_5S-23S ribosomal RNA intergenic spacer, partial sequence**

GAGTTCGCGGGAGAGTAAGTTATTGCCAGGGTTTTTATTTTGTAATTTAAACCTTAAATTTATTTTTTATATTTTTTTAATGTTCATGTTTTTGAATGTTTTATTCAAATAATGTAAAAAATAAAATAGATATTGACATGGATTGAACAAAAGATATATATTATTTTATGTTGCATAAACAAATTGGCAAAATAGAGATGGAAGATAAAAATATGGTCAAAGTAATAAGAGTCTATGGTGAATGCC

**>11_2_Borrelia valaisiana_5S-23S ribosomal RNA intergenic spacer, partial sequence**

GAGTTCGCGGGAGAGTAAGTTATTGCCAGGGTTTTTATTTTGTAATTTAAACCTTAAATTTATTTTTTATATTTTTTTAATGTTTATGTTTTTGAATGTTTTATTCAAATAATGTAAAAAATAAAATAGATATTGACATGGATTGAACAAAAGATATATATTATTTTATGTTGCATAAACAAATTGGCAAAATAGAGATGGAAGATAAAAATATGGTCAAAGTAATAAGAGTCTATGGTGAATGCC

**>12_2_Borrelia valaisiana_5S-23S ribosomal RNA intergenic spacer, partial sequence**

GAGTTCGCGGGAGAGTAAGTTATTGCCAGGGTTTTTATTTTGTAATTTAAACCTTAAATTTATTTTTTATATTTTTATAATGTTCATGTTTTTGAATGTTTTATTCAAATAATGTAAAAAATAAAATAGATATTGACATGGATTGAACAAAAGATATATATTATTTTATGTTGCATAAACAAATTGGCAAAATAGAGATGGAAGATAAAAATATGGTCAAAGTAATAAGAGTCTATGGTGAATGCC

**>38_2_Borrelia valaisiana_5S-23S ribosomal RNA intergenic spacer, partial sequence**

GAGTTCGCGGGAGAGTAAGTTATTGCCAGGGTTTTTATTTTGTAATTTAAACCTTAAATTTATTTTTTATATTTTTTTAATGTTCATGTTTTTGAATGTTTTATTCAAATAATGTAAAAAATAAAATAGATATTGACATGGATTGAACAAAAGATATATATTATTTTATGTTGCATAAACAAATTGGCAAAATAGAGATGGAAGATAAAAATATGGTCAAAGTAATAAGAGTCTATGGTGAATGCC

**>39_2_Borrelia valaisiana_5S-23S ribosomal RNA intergenic spacer, partial sequence**

GAGTTCGCGGGAGAGTAAGTTATTGCCAGGGTTTTTATTTTGTACTTTAAACCTTAAATTTATTTTTTATATTTTTTTAATGTTCATGTTTTTGAATGTTTTATTCAAATAATGTAAAAAATAAAATAGATATTGACATGGATTGAACAAAAGATATATATTATTTTATGTTGCATAAACAAATTGGCAAAATAGAGATGGAAGATAAAAATATGGTCAAAGTAATAAGAGTCTATGGTGAATGCC

**>40_2_Borrelia valaisiana_5S-23S ribosomal RNA intergenic spacer, partial sequence**

GAGTTCGCGGGAGAGTAAGTTATTGCCAGGGTTTTTATTTTGTAATTTAAACCTTAAATTTATTTTTTTAATGTTTATGTTTTTGAATGTTTTATTCAAATAATGTAAAAAATAAAATAGATATTGACATGGATTGAACAAAAGATATATATTATTTTATGTTGCATAAACAAATTGGCAAAATAGAGATGGAAGATAAAAATATGGTCAAAGTAATAAGAGTCTATGGTGAATGCC

**>41_2_Borrelia valaisiana_5S-23S ribosomal RNA intergenic spacer, partial sequence**

GAGTTCGCGGGAGAGTAAGTTATTGCCAGGGTTTTTATTTTGTAATTTAAACCTTAAATTTATTTTTTATATTTTTTTAATGTTCATGTTTTTGAATGTTTTATTCAAATAATGTAAAAAATAAAATAGATATTGACATGGATTGAACAAAAGATATATATTATTTTATGTTGCATAAACAAATTGGCAAAATAGAGATGGAAGATAAAAATATGGTCAAAGTAATAAGAGTCTATGGTGAATGCC

**>5_3_Borrelia valaisiana_5S-23S ribosomal RNA intergenic spacer, partial sequence**

GAGTTCGCGGGAGAGTAAGTTATTGCCAGGGTTTTTATTTTGTAATTTAAACCTTAAATTTATTTTTTATATTTTTTTAATGTTCATGTTTTTGAATGTTTTATTCAAATAATGTAAAAAATAAAATAGATATTGACATGGATTGAACAAAAGATATATATTATTTTATGTTGCATAAACAAATTGGCAAAATAGAGATGGAAGATAAAAATATGGTCAAAGTAATAAGAGTCTATGGTGAATGCC

**>7_3_Borrelia valaisiana_5S-23S ribosomal RNA intergenic spacer, partial sequence**

GAGTTCGCGGGAGAGTAAGTTATTGCCAGGGTTTTTATTTTGTAATTTAAACCTTAAATTTATTTTTTATATTTTTTTAATGTTCATGTTTTTGAATGTTTTATTCAAATAATGTAAAAAATAAAATAGATATTGACATGGATTGAACAAAAGATATATATTATTTTATGTTGCATAAACAAATTGGCAAAATAGAGATGGAAGATAAAAATATGGTCAAAGTAATAAGAGTCTATGGTGAATGCC

**>8_3_Borrelia valaisiana_5S-23S ribosomal RNA intergenic spacer, partial sequence**

GAGTTCGCGGGAGAGTAAGTTATTGCCAGGGTTTTTATTTTGTAATTTAAACCTTAAATTTATTTTTTATATTTTTTTAATGTTCATGTTTTTGAATGTTTTATTCAAATAATGTAAAAAATAAAATAGATATTGACATGGATTGAACAAAAGATATATATTATTTTATGTTGCATAAACAAATTGGCAAAATAGAGATGGAAGATAAAAATATGGTCAAAGTAATAAGAGTCTATGGTGAATGCC

**>15_3_Borrelia valaisiana_5S-23S ribosomal RNA intergenic spacer, partial sequence**

GAGTTCGCGGGAGAGTAAGTTATTGCCAGGGTTTTTATTTTGTAATTTAAACCTTAAATTTATTTTTTATATTTTTTTAATGTTCATGTTTTTGAATGTTTTATTCAAATAATGTAAAAAATAAAATAGATATTGACATGGATTGAACAAAAGATATATATTATTTTATGTTGCATAAACAAATTGGCAAAATAGAGATGGAAGATAAAAATATGGTCAAAGTAATAAGAGTCTATGGTGAATGCC

**>35_5_Borrelia valaisiana_5S-23S ribosomal RNA intergenic spacer, partial sequence**

GAGTTCGCGGGAGAGTAAGTTATTGCCAGGGTTTTTATTTTGTACTTTAAACCTTAAATTTATTTTTTATATTTTTTTAATGTTCATGTTTTTGAATGTTTTATTCAAATAATGTAAAAAATAAAATAGATATTGACATGGATTGAACAAAAGATATATATTATTTTATGTTGCATAAACAAATTGGCAAAATAGAGATGGAAGATAAAAATATGGTCAAAGTAATAAGAGTCTATGGTGAATGCC

**>11_6_Borrelia valaisiana_5S-23S ribosomal RNA intergenic spacer, partial sequence**

GAGTTCGCGGGAGAGTAAGTTATTGCCAGGGTTTTTATTTTGTAATTTAAACCTTAAATTTATTTTTTATATTTTTTTAATGTTCATGTTTTTGAATGTTTTATTCAAATAATGTAAAAAATAAAATAGATATTGACATGGATTGAACAAAAGATATATATTATTTTATGTTGCATAAACAAATTGGCAAAATAGAGATGGAAGATAAAAATATGGTCAAAGTAATAAGAGTCTATGGTGAATGCC

**>32_16_Borrelia valaisiana_5S-23S ribosomal RNA intergenic spacer, partial sequence**

GAGTTCGCGGGAGAGTAAGTTATTGCCAGGGTTTTTATTTTGTAATTTAAACCTTAAATTTATTTTTTATATTTTTTTAATGTTCATGTTTTTGAATGTTTTATTCAAATAATGTAAAAAATAAAATAGATATTGACATGGATTGAACAAAAGATATATATTATTTTATGTTGCATAAACAAATTGGCAAAATAGAGATGGAAGATAAAAATATGGTCAAAGTAATAAGAGTCTATGGTGAATGCC

**>34_16_Borrelia valaisiana_5S-23S ribosomal RNA intergenic spacer, partial sequence**

GAGTTCGCGGGAGAGTAAGTTATTGCCAGGGTTTTTATTTTGTAATTTAAACCTTAAATTTATTTTTTATATTTTTTTAATGTTCATGTTTTTGAATGTTTTATTCAAATAATGTAAAAAATAAAATAGATATTGACATGGATTGAACAAAAGATATATATTATTTTATGTTGCATAAACAAATTGGCAAAATAGAGATGGAAGATAAAAATATGGTCAAAGTAATAAGAGTCTATGGTGAATGCC

**>39_16_Borrelia valaisiana_5S-23S ribosomal RNA intergenic spacer, partial sequence**

GAGTTCGCGGGAGAGTAAGTTATTGCCAGGGTTTTTATTTTGTACTTTAAACCTTAAATTTATTTTTTATATTTTTTTAATGTTCATGTTTTTGAATGTTTTATTCAAATAATGTAAAAAATAAAATAGATATTGACATGGATTGAACAAAAGATATATATTATTTTATGTTGCATAAACAAATTGGCAAAATAGAGATGGAAGATAAAAATATGGTCAAAGTAATAAGAGTCTATGGTGAATGCC

**>3_17_Borrelia valaisiana_5S-23S ribosomal RNA intergenic spacer, partial sequence**

GAGTTCGCGGGAGAGTAAGTTATTGCCAGGGTTTTTATTTTGTACTTTAAACCTTAAATTTATTTTTTATATTTTTTTAATGTTCATGTTTTTGAATGTTTTATTCAAATAATGTAAAAAATAAAATAGATATTGACATGGATTGAACAAAAGATATATATTATTTTATGTTGCATAAACAAATTGGCAAAATAGAGATGGAAGATAAAAATATGGTCAAAGTAATAAGAGTCTATGGTGAATGCC

**>14_17_Borrelia valaisiana_5S-23S ribosomal RNA intergenic spacer, partial sequence**

GAGTTCGCGGGAGAGTAAGTTATTGCCAGGGTTTTTATTTTGTAATTTAAACCTTAAATTTATTTTTTATATTTTTTTAATGTTCATGTTTTTGAATGTTTTATTCAAATAATGTAAAAAATAAAATAGATATTGACATGGATTGAACAAAAGATATATATTATTTTATGTTGCATAAACAAATTGGCAAAATAGAGATGGAAGATAAAAATATGGTCAAAGTAATAAGAGTCTATGGTGAATGCC

**>39_18_Borrelia valaisiana_5S-23S ribosomal RNA intergenic spacer, partial sequence**

GAGTTCGCGGGAGAGTAAGTTATTGCCAGGGTTTTTATTTTGTACTTTAAACCTTAAATTTATTTTTTATATTTTTTTAATGTTCATGTTTTTGAATGTTTTATTCAAATAATGTAAAAAATAAAATAGATATTGACATGGATTGAACAAAAGATATATATTATTTTATGTTGCATAAACAAATTGGCAAAATAGAGATGGAAGATAAAAATATGGTCAAAGTAATAAGAGTCTATGGTGAATGCC

**>41_18_Borrelia valaisiana_5S-23S ribosomal RNA intergenic spacer, partial sequence**

GAGTTCGCGGGAGAGTAAGTTATTGCCAGGGTTTTTATTTTGTAATTTAAACCTTAAATTTATTTTTTATATTTTTTTAATGTTCATGTTTTTGAATGTTTTATTCAAATAATGTAAAAAATAAAATAGATATTGACATGGATTGAACAAAAGATATATATTATTTTATGTTGCATAAACAAATTGGCAAAATAGAGATGGAAGATAAAAATATGGTCAAAGTAATAAGAGTCTATGGTGAATGCC

**>7_21_Borrelia valaisiana_5S-23S ribosomal RNA intergenic spacer, partial sequence**

GAGTTCGCGGGAGAGTAAGTTATTGCCAGGGTTTTTATTTTGTACTTTAAACCTTAAATTTATTTTTTATATTTTTTTAATGTTCATGTTTTTGAATGTTTTATTCAAATAATGTAAAAAATAAAATAGATATTGACATGGATTGAACAAAAGATATATATTATTTTATGTTGCATAAACAAATTGGCAAAATAGAGATGGAAGATAAAAATATGGTCAAAGTAATAAGAGTCTATGGTGAATGCC

**>8_21_Borrelia valaisiana_5S-23S ribosomal RNA intergenic spacer, partial sequence**

GAGTTCGCGGGAGAGTAAGTTATTGCCAGGGTTTTTATTTTGTAATTTAAACCTTAAATTTATTTTTTATATTTTTTTAATGTTCATGTTTTTGAATGTTTTATTCAAATAATGTAAAAAATAAAATAGATATTGACATGGATTGAACAAAAGATATATATTATTTTATGTTGCATAAACAAATTGGCAAAATAGAGATGGAAGATAAAAATATGGTCAAAGTAATAAGAGTCTATGGTGAATGCC

**>28_24_Borrelia valaisiana_5S-23S ribosomal RNA intergenic spacer, partial sequence**

GAGTTCGCGGGAGAGTAAGTTATTGCCAGGGTTTTTATTTTGTACTTTAAACCTTAAATTTATTTTTTATATTTTTTTAATGTTCATGTTTTTGAATGTTTTATTCAAATAATGTAAAAAATAAAATAGATATTGACATGGATTGAACAAAAGATATATATTATTTTATGTTGCATAAACAAATTGGCAAAATAGAGATGGAAGATAAAAATATGGTCAAAGTAATAAGAGTCTATGGTGAATGCC

**>29_24_Borrelia valaisiana_5S-23S ribosomal RNA intergenic spacer, partial sequence**

GAGTTCGCGGGAGAGTAAGTTATTGCCAGGGTTTTTATTTTGTAATTTAAACCTTAAATTTATTTTTTATATTTTTTTAATGTTCATGTTTTTGAATGTTTTATTCAAATAATGTAAAAAATAAAATAGATATTGACATGGATTGAACAAAAGATATATATTATTTTATGTTGCATAAACAAATTGGCAAAATAGAGATGGAAGATAAAAATATGGTCAAAGTAATAAGAGTCTATGGTGAATGCC

**>30_24_Borrelia valaisiana_5S-23S ribosomal RNA intergenic spacer, partial sequence**

GAGTTCGCGGGAGAGTAAGTTATTGCCAGGGTTTTTATTTTGTACTTTAAACCTTAAATTTATTTTTTATATTTTTTTAATGTTCATGTTTTTGAATGTTTTATTCAAATAATGTAAAAAATAAAATAGATATTGACATGGATTGAACAAAAGATATATATTATTTTATGTTGCATAAACAAATTGGCAAAATAGAGATGGAAGATAAAAATATGGTCAAAGTAATAAGAGTCTATGGTGAATGCC

**>32_24_Borrelia valaisiana_5S-23S ribosomal RNA intergenic spacer, partial sequence**

GAGTTCGCGGGAGAGTAAGTTATTGCCAGGGTTTTTATTTTGTACTTTAAACCTTAAATTTATTTTTTATATTTTTTTAATGTTCATGTTTTTGAATGTTTTATTCAAATAATGTAAAAAATAAAATAGATATTGACATGGATTGAACAAAAGATATATATTATTTTATGTTGCATAAACAAATTGGCAAAATAGAGATGGAAGATAAAAATATGGTCAAAGTAATAAGAGTCTATGGTGAATGCC

**>21_25_Borrelia valaisiana_5S-23S ribosomal RNA intergenic spacer, partial sequence**

GAGTTCGCGGGAGAGTAAGTTATTGCCAGGGTTTTTATTTTGTACTTTAAACCTTAAATTTATTTTTTATATTTTTTTAATGTTCATGTTTTTGAATGTTTTATTCAAATAATGTAAAAAATAAAATAGATATTGACATGGATTGAACAAAAGATATATATTATTTTATGTTGCATAAACAAATTGGCAAAATAGAGATGGAAGATAAAAATATGGTCAAAGTAATAAGAGTCTATGGTGAATGCC

**>39_25_Borrelia valaisiana_5S-23S ribosomal RNA intergenic spacer, partial sequence**

GAGTTCGCGGGAGAGTAAGTTATTGCCAGGGTTTTTATTTTGTACTTTAAACCTTAAATTTATTTTTTATATTTTTTTAATGTTCATGTTTTTGAATGTTTTATTCAAATAATGTAAAAAATAAAATAGATATTGACATGGATTGAACAAAAGATATATATTATTTTATGTTGCATAAACAAATTGGCAAAATAGAGATGGAAGATAAAAATATGGTCAAAGTAATAAGAGTCTATGGTGAATGCC

**>13_27_Borrelia valaisiana_5S-23S ribosomal RNA intergenic spacer, partial sequence**

GAGTTCGCGGGAGAGTAAGTTATTGCCAGGGTTTTTATTTTGTACTTTAAACCTTAAATTTATTTTTTATATTTTTTTAATGTTCATGTTTTTGAATGTTTTATTCAAATAATGTAAAAAATAAAATAGATATTGACATGGATKGAACAAAAGATATATATTATTTTATGTTGCATAAACAAATTGGCAAAATAGAGATGGAAGATAAAAATATGGTCAAAGTAATAAGAGTCTATGGTGAATGCC

**>20_27_Borrelia afzelii_5S-23S ribosomal RNA intergenic spacer, partial sequence**

GAGTTCGCGGGAGAGTAAGTTATTGCCAGGGTTTTTATTTTATACTTTAAACCTTGAATTTATTTTTTAAATGTGTTTATATTATTTGAATAAAACATTCAAATAATATAAAAAATAATATATATATTGACATGGATTAAACAAAGATATATATTATTTTATGTTGTATGAACAAATTGGCAAAATAGAGATGGAAGATAAAAATATGGTCAAAGTAATAAGAGTCTATGGTGAATGCC

**>8_1_Borrelia afzelii_5S-23S ribosomal RNA intergenic spacer, partial sequence**

GAGTTCGCGGGAGAGTAAGTTATTGCCAGGGTTTTTATTTTATACTTTAAACCTTGAATTTATTTTTTAAATGTGTTTATATTATTTGAATAAAACATTCAAATAATATAAAAAATAATATATATATTGACATGGATTAAACAAAGATATATATTATTTTATGTTGTATGAACAAATTGGCAAAATAGAGATGGAAGATAAAAATATGGTCAAAGTAATAAGAGTCTATGGTGAATGCC

**>30_3_Borrelia afzelii_5S-23S ribosomal RNA intergenic spacer, partial sequence**

GAGTTCGCGGGAGAGTAAGTTATTGCCAGGGTTTTTATTTTATACTTTAAACCTTAAATTTATTTTTTAAATGTTTATATTATTTGAATGTTTTATTCAAATAATATAAAAAATAATATATATATTGACATGGATTAAACAAAGATATATATTATTTTATGTTGTATAAACAAATTGGCAAAATAGAGATGGAAGATAAAAATATGGTCAAAGTAATAAGAGTCTATGGTGAATGCC

**>1_4_Borrelia afzelii_5S-23S ribosomal RNA intergenic spacer, partial sequence**

GAGTTCGCGGGAGAGTAAGTTATTGCCAGGGTTTTTATTTTATACTTTAAACCTTGAATTTATTTTTTAAATGTTTATATTATTTGAATAAAACATTCAAATAATATAAAAAATAATATATATATTGACATGGATTAAACAAAGATATATATTATTTTATGTTGTATAAACAAATTGGCAAAATAGAGATGGAAGATAAAAATATGGTCAAAGTAATAAGAGTCTATGGTGAATGCC

**>23_4_Borrelia afzelii_5S-23S ribosomal RNA intergenic spacer, partial sequence**

GAGTTCGCGGGAGAGTAAGTTATTGCCAGGGTTTTTATTTTATACTTTAAACCTTGAATTTATTTTTTAAATGTGTTTATATTATTTGAATAAAACATTCAAATAATATAAAAAATAATATATATATTGACATGGATTAAACAAAGATATATATTATTCTATGTTGTATGAACAAATTGGCAAAATAGAGATGGAAGATAAAAATATGGTCAAAGTAATAAGAGTCTATGGTGAATGCC

**>26_6_Borrelia afzelii_5S-23S ribosomal RNA intergenic spacer, partial sequence**

GAGTTCGCGGGAGAGTAAGTTATTGCCAGGGTTTTTATTTTATACTTTAAACCTTGAATTTATTTTTTAAATGTTTATATTATTTGAATAAAACATTCAAATAATATAAAAAATAATATATATATTGACATGGATTAAACAAAGATATATATTATTCTATGTTGTATAAACAAATTGGCAAAATAGAGATGGAAGATAAAAATATGGTCAAAGTAATAAGAGTCTATGGTGAATGCC

**>15_7_Borrelia afzelii_5S-23S ribosomal RNA intergenic spacer, partial sequence**

GAGTTCGCGGGAGAGTAAGTTATTGCCAGGGTTTTTATTTTATACTTTAAACCTTGAATTTATTTTTTAAATGTTTATATTATTTGAATAAAACATTCAAATAATATAAAAAATAATATATATATTGACATGGATTAAACAAAGATATATATTATTCTATGTTGTATAAACAAATTGGCAAAATAGAGATGGAAGATAAAAATATGGTCAAAGTAATAAGAGTCTATGGTGAATGCC

**>39_8_Borrelia afzelii_5S-23S ribosomal RNA intergenic spacer, partial sequence**

GAGTTCGCGGGAGAGTAAGTTATTGCCAGGGTTTTTATTTTATACTTTAAACCTTGAATTTATTTTTTAAATGTTTATATTATTTGAATAAAACATTCAAATAATATAAAAAATAATATATATATTGACATGGATTAAACAAAGATATATATTATTCTATGTTGTATAAACAAATTGGCAAAATAGAGATGGAAGATAAAAATATGGTCAAAGTAATAAGAGTCTATGGTGAATGCC

**>23_10_Borrelia afzelii_5S-23S ribosomal RNA intergenic spacer, partial sequence**

GAGTTCGCGGGAGAGTAAGTTATTGCCAGGGTTTTTATTTTATACTTTAAACCTTGAATTTATTTTTTAAATGTTTATATTATTTGAATAAAACATTCAAATAATATAAAAAATAATATATATATTGACATGGATTAAACAAAGATATATATTATTTTATGTTGTATAAACAAATTGGCAAAATAGAGATGGAAGATAAAAATATGGTCAAAGTAATAAGAGTCTATGGTGAATGCC

**>30_11_Borrelia afzelii_5S-23S ribosomal RNA intergenic spacer, partial sequence**

GAGTTCGCGGGAGAGTAAGTTATTGCCAGGGTTTTTATTTTATACTTTAAACCTTGAATTTATTTTTTAAATGTTTATATTATTTGAATGTTTTATTCAAATAATATAAAAAATAATATATATATTGACATGGATTAAACAAAGATATATATTATTCTATGTTGTATAAACAAATTGGCAAAATAGAGATGGAAGATAAAAATATGGTCAAAGTAATAAGAGTCTATGGTGAATGCC

**>16_12_Borrelia afzelii_5S-23S ribosomal RNA intergenic spacer, partial sequence**

GAGTTCGCGGGAGAGTAAGTTATTGCCAGGGTTTTTATTTTATACTTTAAACCTTGAATTTATTTTTTAAATGTTTATATTATTTGAATAAAACATTCAAATAATATAAAAAATAATATATATATTGACATGGATTAAACAAAGATATATATTATTTTATGTTGTATAAACAAATTGGCAAAATAGAGATGGAAGATAAAAATATGGTCAAAGTAATAAGAGTCTATGGTGAATGCC

**>38_13_Borrelia afzelii_5S-23S ribosomal RNA intergenic spacer, partial sequence**

GAGTTCGCGGGAGAGTAAGTTATTGCCAGGGTTTTTATTTTATACTTTAAACCTTGAATTTATTTTTTAAATGTTTATATTATTTGAATAAAACATTCAAATAATATAAAAAATAATATATATATTGACATGGATTAAACAAAGATATATATTATTTTATGTTGTATAAACAAATTGGCAAAATAGAGATGGAAGATAAAAATATGGTCAAAGTAATAAGAGTCTATGGTGAATGCC

**>35_16_Borrelia afzelii_5S-23S ribosomal RNA intergenic spacer, partial sequence**

GAGTTCGCGGGAGAGTAAGTTATTGCCAGGGTTTTTATTTTATACTTTAAACCTTGAATTTATTTTTTAAATGTGTTTATATTATTTGAATAAAACATTCAAATAATATAAAAAATAATATATATATTGACATGGATTAAACAAAGATATATATTATTCTATGTTGTATGAACAAATTGGCAAAATAGAGATGGAAGATAAAAATATGGTCAAAGTAATAAGAGTCTATGGTGAATGCC

**>16_17_Borrelia afzelii_5S-23S ribosomal RNA intergenic spacer, partial sequence**

GAGTTCGCGGGAGAGTAAGTTATTGCCAGGGTTTTTATTTTATACTTTAAACCTTGAATTTATTTTTTAAATGTTTATATTATTTGAATAAAACATTCAAATAATATAAAAAATAATATATATATTGACATGGATTAAACAAAGATATATATTATTTTATGTTGTATAAACAAATTGGCAAAATAGAGATGGAAGATAAAAATATGGTCAAAGTAATAAGAGTCTATGGTGAATGCC

**>33_18_Borrelia afzelii_5S-23S ribosomal RNA intergenic spacer, partial sequence**

GAGTTCGCGGGAGAGTAAGTTATTGCCAGGGTTTTTATTTTATACTTTAAATCTTGAATTTATTTTTTAAATGTTTATATTATTTGAATGTTTTATTCAAATAATATAAAAAATAATATATATATTGACATGGATTAAACAAAGATATATATTATTCTATGTTGTATAAACAAATTGGCAAAATAGAGATGGAAGATAAAAATATGGTCAAAGTAATAAGAGTCTATGGTGAATGCC

**>30_19_Borrelia afzelii_5S-23S ribosomal RNA intergenic spacer, partial sequence**

GAGTTCGCGGGAGAGTAAGTTATTGCCAGGGTTTTTATTTTATACTTTAAACCTTGAATTTATTTTTTAAATGTTTATATTATTTGAATAAAACATTCAAATAATATAAAAAATAATATATATATTGACATGGATTAAACAAAGATATATATTATT CTATGTTGTATGAACAAATTGGCAAAATAGAGATGGAAGATAAAAATATGGTCAAAGTAATAAGAGTCTATGGTGAATGCC

**>31_19_Borrelia afzelii_5S-23S ribosomal RNA intergenic spacer, partial sequence**

GAGTTCGCGGGAGAGTAAGTTATTGCCAGGGTTTTTATTTTATACTTTAAACCTTGAATTTATTTTTTAAATGTTTATATTATTTGAATAAAACATTCAAATAATATAAAAAATAATATATATATTGACATGGATTAAACAAAGATATATATTATTCTATGTTGTATGAACAAATTGGCAAAATAGAGATGGAAGATAAAAATATGGTCAAAGTAATAAGAGTCTATGGTGAATGCC

**>38_19_Borrelia afzelii_5S-23S ribosomal RNA intergenic spacer, partial sequence**

GAGTTCGCGGGAGAGTAAGTTATTGCCAGGGTTTTTATTTTATACTTTAAATCTTGAATTTATTTTTTAAATGTTTATATTATTTGAATGTTTTATTCAAATAATATAAAAAATAATATATATATTGACATGGATTAAACAAAGATATATATTATTCTATGTTGTATAAACAAATTGGCAAAATAGAGATGGAAGATAAAAATATGGTCAAAGTAATAAGAGTCTATGGTGAATGCC

**>5_22_Borrelia afzelii_5S-23S ribosomal RNA intergenic spacer, partial sequence**

GAGTTCGCGGGAGAGTAAGTTATTGCCAGGGTTTTTATTTTATACTTTAAACCTTAAATTTATTTTTTAAATGTTTATATTATTTGAATGTTTTATTCAAATAATATAAAAAATAATATATATATTGACATGGATTAAACAAAGATATATATTATTTTATGTTGTATAAACAAATTGGCAAAATAGAGATGGAAGATAAAAATATGGTCAAAGTAATAAGAGTCTATGGTGAATGCC

**>12_22_Borrelia afzelii_5S-23S ribosomal RNA intergenic spacer, partial sequence**

GAGTTCGCGGGAGAGTAAGTTATTGCCAGGGTTTTTATTTTATACTTTAAATCTTGAATTTATTTTTTAAATGTTTATATTATTTGAATGTTTTATTCAAATAATATAAAAAATAATATATATATTGACATGGATTAAACAAAGATATATATTATTCTATGTTGTATAAACAAATTGGCAAAATAGAGATGGAAGATAAAAATATGGTCAAAGTAATAAGAGTCTATGGTGAATGCC

**>4_23_Borrelia afzelii_5S-23S ribosomal RNA intergenic spacer, partial sequence**

GAGTTCGCGGGAGAGTAAGTTATTGCCAGGGTTTTTATTTTATACTTTAAACCTTGAATTTATTTTTTAAATGTTTATATTATTTGAATAAAACATTCAAATAATATAAAAAATAATATATATATTGACATGGATTAAACAAAGATATATATTATTCTATGTTGTATGAACAAATTGGCAAAATAGAGATGGAAGATAAAAATATGGTCAAAGTAATAAGAGTCTATGGTGAATGCC

**>33_23_Borrelia afzelii_5S-23S ribosomal RNA intergenic spacer, partial sequence**

GAGTTCGCGGGAGAGTAAGTTATTGCCAGGGTTTTTATTTTATACTTTAAACCTTGAATTTATTTTTTAAATGTTTATATTATTTGAATAAAACATTCAAATAATATAAAAAATAATATATATATTGACATGGATTAAACAAAGATATATATTATTCTATGTTGTATGAACAAATTGGCAAAATAGAGATGGAAGATAAAAATATGGTCAAAGTAATAAGAGTCTATGGTGAATGCC

**>30_25_Borrelia afzelii_5S-23S ribosomal RNA intergenic spacer, partial sequence**

GAGTTCGCGGGAGAGTAAGTTATTGCCAGGGTTTTTATTTTATACTTTAAATCTTGAATTTATTTTTTAAATGTTTATATTATTTGAATGTTTTATTCAAATAATATAAAAAATAATATATATATTGACATGGATTAAACAAAGATATATATTATTCTATGTTGTATAAACAAATTGGCAAAATAGAGATGGAAGATAAAAATATGGTCAAAGTAATAAGAGTCTATGGTGAATGCC

**>31_25_Borrelia afzelii_5S-23S ribosomal RNA intergenic spacer, partial sequence**

GAGTTCGCGGGAGAGTAAGTTATTGCCAGGGTTTTTATTTTATACTTTAAACCTTGAATTTATTTTTTAAATGTTTATATTATTTGAATAAAACATTCAAATAATATAAAAAATAATATATATATTGACATGGATTAAACAAAGATATATATTATTCTATGTTGTATGAACAAATTGGCAAAATAGAGATGGAAGATAAAAATATGGTCAAAGTAATAAGAGTCTATGGTGAATGCC

**>1_26_Borrelia afzelii_5S-23S ribosomal RNA intergenic spacer, partial sequence**

GAGTTCGCGGGAGAGTAAGTTATTGCCAGGGTTTTTATTTTATACTTTAAACCTTGAATTTATTTTTTAAATGTTTATATTATTTGAATAAAACATTCAAATAATATAAAAAATAATATATATATTGACATGGATTAAACAAAGATATATATTATTCTATGTTGTATAAACAAATTGGCAAAATAGAGATGGAAGATAAAAATATGGTCAAAGTAATAAGAGTCTATGGTGAATGCC

**>8_26_Borrelia afzelii_5S-23S ribosomal RNA intergenic spacer, partial sequence**

GAGTTCGCGGGAGAGTAAGTTATTGCCAGGGTTTTTATTTTATACTTTAAACCTTGAATTTATTTTTTAAATGTTTATATTATTTGAATAAAACATTCAAATAATATAAAAAATAATATATATATTGACATGGATTAAACAAAGATATATATTATTCTATGTTGTATGAACAAATTGGCAAAATAGAGATGGAAGATAAAAATATGGTCAAAGTAATAAGAGTCTATGGTGAATGCC

**>9_26_Borrelia afzelii_5S-23S ribosomal RNA intergenic spacer, partial sequence**

GAGTTCGCGGGAGAGTAAGTTATTGCCAGGGTTTTTATTTTATACTTTAAACCTTGAATTTATTTTTTAAATGTTTATATTATTTGAATAAAACATTCAAATAATATAAAAAATAATATATATATTGACATGGATTAAACAAAGATATATATTATTTTATGTTGTATAAACAAATTGGCAAAATAGAGATGGAAGATAAAAATATGGTCAAAGTAATAAGAGTCTATGGTGAATGCC

**>20_26_Borrelia afzelii_5S-23S ribosomal RNA intergenic spacer, partial sequence**

GAGTTCGCGGGAGAGTAAGTTATTGCCAGGGTTTTTATTTTATACTTTAAATCTTGAATTTATTTTTTAAATGTTTATATTATTTGAATGTTTTATTCAAATAATATAAAAAATAATATATATATTGACATGGATTAAACAAAGATATATATTATTCTATGTTGTATAAACAAATTGGCAAAATAGAGATGGAAGATAAAAATATGGTCAAAGTAATAAGAGTCTATGGTGAATGCC

**>41_26_Borrelia afzelii_5S-23S ribosomal RNA intergenic spacer, partial sequence**

GAGTTCGCGGGAGAGTAAGTTATTGCCAGGGTTTTTATTTTATACTTTAAACCTTAAATTTATTTTTTAAATGTTTATATTATTTGAATGTTTTATTCAAATAATATAAAAAATAATATATATATTGACATGGATTAAACAAAGATATATATTATTTTATGTTGTATAAACAAATTGGCAAAATAGAGATGGAAGATAAAAATATGGTCAAAGTAATAAGAGTCTATGGTGAATGCC

**>11_3_Borrelia afzelii_5S-23S ribosomal RNA intergenic spacer, partial sequence**

GAGTTCGCGGGAGAGTAAGTTATTGCCAGGGTTTTTATTTTATACTTTAAACCTTAAATTTATTTTTTAAATGTTTATATTATTTGAATGTTTTATTCAAATAATATAAAAAATAATATATATATTGACATGGATTAAACAAAGATATATATTATTTTATGTTGTATAAACAAATTGGCAAAATAGAGATGGAAGATAAAAATATGGTCAAAGTAATAAGAGTCTATGGTGAATGCC

**>13_3_Borrelia garinii_5S-23S ribosomal RNA intergenic spacer, partial sequence**

GAGTTCGCGGGAGAGTAAGTTATTGCCAGGGTTTTTATTTTATACTTTAAACATTGATTTTATTTTTTATGTTTTTAGATGTTTATGTTTTTGAATGTTTTATTCAAATAATATAAAAAATAAAATATATATTGACATGGATTAAACAAAGATATATATTATTCTATGTTGTATAAACAAATTGGCAAAATAGAGATGGAAGATAAAAATATGGTCAAAGTAATAAGAGTCTATGGTGAATGCC

**>28_4_Borrelia garinii_5S-23S ribosomal RNA intergenic spacer, partial sequence**

GAGTTCGCGGGAGAGTAAGTTGTTGCCAGGGTTTTTGTTTTATACTTTAAACATTGATTTTATTTTTTATGTTTTTAGATGTTCATGTTTTTGAATGTTTTATTCAAATAATATAAAAAATAAAATATATATTGACATGGATTAAACAAAGATATATATTATTCTATGTTGTATAAACAAATTGGCAAAATAGAGATGGAAGATAAAAATATGGTCAAAGTAATAAGAGTCTATGGTGAATGCC

**>9_7_Borrelia garinii_5S-23S ribosomal RNA intergenic spacer, partial sequence**

GAGTTCGCGGGAGAGTAAGTTATTGCCAGGGTTTTTATTTTATACTTTAAACATTGATTTTATTTTTTATGTTTTTAGATGTTCATGTTTTTGAATGTTTTATTCGAATAATATAAAAAATAAAATATATATTGACATGGATTAAACAAAGATATATATTATTCTATGTTGTATAAACAAATTGGCAAAATAGAGATGGAAGATAAAAATATGGTCAAAGTAATAAGAGTCTATGGTGAATGCC

**>10_7_Borrelia garinii_5S-23S ribosomal RNA intergenic spacer, partial sequence**

GAGTTCGCGGGAGAGTAAGTTATTGCCAGGGTTTTTATTTTATACTTTAAACATTGATTTTATTTTTTATGTTTTTAGATGTTCATGTTTTTGAATGTTTTATTCGAATAATATAAAAAATAAAATATATATTGACATGGATTAAACAAAGATATATATTATTCTATGTTGTATAAACAAATTGGCAAAATAGAGATGGAAGATAAAAATATGGTCAAAGTAATAAGAGTCTATGGTGAATGCC

**>20_7_Borrelia garinii_5S-23S ribosomal RNA intergenic spacer, partial sequence**

GAGTTCGCGGGAGAGTAAGTTATTGCCAGGGTTTTTATTTTATACTTTAAACATTGATTTTATTTTTTATGTTTTTAGATGTTCATGTTTTTCAATGTTTTATTCAAATAATATAAAAAATAAAATATATATTGACATGGATTAAACAAAGATATATATTATTCTATGTTGTATAAACAAATTGGCAAAATAGAGATGGAAGATAAAAATATGGTCAAAGTAATAAGAGTCTATGGTGAATGCC

**>44_7_Borrelia garinii_5S-23S ribosomal RNA intergenic spacer, partial sequence**

GAGTTCGCGGGAGAGTAAGTTATTGCCAGGGTTTTTCTTTTATACTTTAAACATTGATTTTATTTTTTATGTTTTTAGATGTTTATATTATTTGAATGTTTTATTCAAATAATATAAAAAATAAAATATATATATTGACATGGATTAAACAAAGATATATATTATTCTATGTTGTATAAACAAATTGGCAAAATAGAGATGGAAGATAAAAATATGGTCAAAGTAATAAGAGTCTATGGTGAATGCC

**>3_8_Borrelia garinii_5S-23S ribosomal RNA intergenic spacer, partial sequence**

GAGTTCGCGGGAGAGTAAGTTATTGCCAGGGTTTTTATTTTATACTTTAAACATTGATTTTATTTTTTATGTTTTTAGATGTTCATGTTTTTGAATGTTTTATTCGAATAATATAAAAAATAAAATATATATATTGACATGGATTAAACAAAGATATATATTATTCTATGTTGTATAAACAAATTGGCAAAATAGAGATGGAAGATAAAAATATGGTCAAAGTAATAAGAGTCTATGGTGAATGCC

**>5_8_Borrelia garinii_5S-23S ribosomal RNA intergenic spacer, partial sequence**

GAGTTCGCGGGAGAGTAAGTTATTGCCAGGGTTTTTCTTTTATACTTTAAACATTGATTTTATTTTTTATGTTTTTAGATGTTTATATTATTTGAATGTTTTATTCAAATAATATAAAAAATAAAATATATATATTGACATGGATTAAACAAAGATATATATTATTCTATGTTGTATAAACAAATTGGCAAAATAGAGATGGAAGATAAAAATATGGTCAAAGTAATAAGAGTCTATGGTGAATGCC

**>7_8_Borrelia garinii_5S-23S ribosomal RNA intergenic spacer, partial sequence**

GAGTTCGCGGGAGAGTAAGTTATTGCCAGGGTTTTTATTTTATACTTTAAACATTGATTTTATTTTTTATGTTTTTAGATGTTCATGTTTTTGAATGTTTTATTCGAATAATATAAAAAATAAAATATATATATTGACATGGATTAAACAAAGATATATATTATTCTATGTTGTATAAACAAATTGGCAAAATAGAGATGGAAGATAAAAATATGGTCAAAGTAATAAGAGTCTATGGTGAATGCC

**>8_8_Borrelia garinii_5S-23S ribosomal RNA intergenic spacer, partial sequence**

GAGTTCGCGGGAGAGTAAGTTATTGCCAGGGTTTTTATTTTATACTTTAAACATTGATTTTATTTTTTATGTTTTTAGATGTTCATGTTTTTGAATGTTTTATTCGAATAATATAAAAAATAAAATATATATATTGACATGGATTAAACAAAGATATATATTATTCTATGTTGTATAAACAAATTGGCAAAATAGAGATGGAAGATAAAAATATGGTCAAAGTAATAAGAGTCTATGGTGAATGCC

**>9_8_Borrelia garinii_5S-23S ribosomal RNA intergenic spacer, partial sequence**

GAGTTCGCGGGAGAGTAAGTTATTGCCAGGGTTTTTATTTTATACTTTAAACATTGATTTTATTTTTTATGTTTTTAGATGTTCATGTTTTTGAATGTTTTATTCGAATAATATAAAAAATAAAATATATATATTGACATGGATTAAACAAAGATATATATTATTCTATGTTGTATAAACAAATTGGCAAAATAGAGATGGAAGATAAAAATATGGTCAAAGTAATAAGAGTCTATGGTGAATGCC

**>10_8_Borrelia garinii_5S-23S ribosomal RNA intergenic spacer, partial sequence**

GAGTTCGCGGGAGAGTAAGTTATTGCCAGGGTTTTTCTTTTATACTTTAAACATTGATTTTATTTTTTATGTTTTTAGATGTTTATATTATTTGAATGTTTTATTCAAATAATATAAAAAATAAAATATATATATTGACATGGATTAAACAAAGATATATATTATTCTATGTTGTATAAACAAATTGGCAAAATAGAGATGGAAGATAAAAATATGGTCAAAGTAATAAGAGTCTATGGTGAATGCC

**>11_8_Borrelia garinii_5S-23S ribosomal RNA intergenic spacer, partial sequence**

GAGTTCGCGGGAGAGTAAGTTATTGCCAGGGTTTTTCTTTTATACTTTAAACATTGATTTTATTTTTTATGTTTTTAGATGTTTATATTATTTGAATGTTTTATTCAAATAATATAAAAAATAAAATATATATATTGACATGGATTAAACAAAGATATATATTATTCTATGTTGTATAAACAAATTGGCAAAATAGAGATGGAAGATAAAAATATGGTCAAAGTAATAAGAGTCTATGGTGAATGCC

**>1_9_Borrelia garinii_5S-23S ribosomal RNA intergenic spacer, partial sequence**

GAGTTCGCGGGAGAGTAAGTTATTGCCAGGGTTTTTATTTTATACTTTAAACATTGATTTTATTTTTTATGTTTTTAGATGTTCATGTTTTTGAATGTTTTATTCGAATAATATAAAAAATAAAATATATATTGACATGGATTAAACAAAGATATATATTATTCTATGTTGTATAAACAAATTGGCAAAATAGAGATGGAAGATAAAAATATGGTCAAAGTAATAAGAGTCTATGGTGAATGCC

**>6_9_Borrelia garinii_5S-23S ribosomal RNA intergenic spacer, partial sequence**

GAGTTCGCGGGAGAGTAAGTTATTGCCAGGGTTTTTCTTTTATACTTTAAACATTGATTTTATTTTTTATGTTTTTAGATGTTTATATTATTTGAATGTTTTATTCAAATAATATAAAAAATAAAATATATATATTGACATGGATTAAACAAAGATATATATTATTCTATGTTGTATAAACAAATTGGCAAAATAGAGATGGAAGATAAAAATATGGTCAAAGTAATAAGAGTCTATGGTGAATGCC

**>7_9_Borrelia garinii_5S-23S ribosomal RNA intergenic spacer, partial sequence**

GAGTTCGCGGGAGAGTAAGTTATTGCCAGGGTTTTTATTTTATACTTTAAACATTGATTTTATTTTTTATGTTTTTAGATGTTCATGTTTTTGAATGTTTTATTCGAATAATATAAAAAATAAAATATATATTGACATGGATTAAACAAAGATATATATTATTCTATGTTGTATAAACAAATTGGCAAAATAGAGATGGAAGATAAAAATATGGTCAAAGTAATAAGAGTCTATGGTGAATGCC

**>12_9_Borrelia garinii_5S-23S ribosomal RNA intergenic spacer, partial sequence**

GAGTTCGCGGGAGAGTAAGTTATTGCCAGGGTTTTTATTTTATACTTTAAACATTGATTTTATTTTTTATGTTTTTAGATGTTTATATTATTTGAATGTTTTATTCAAATAATATAAAAAATAAAATATATATATTGACATGGATTAAACAAAGATATATATTATTCTATGTTGTATAAACAAATTGGCAAAATAGAGATGGAAGATAAAAATATGGTCAAAGTAATAAGAGTCTATGGTGAATGCC

**>13_9_Borrelia garinii_5S-23S ribosomal RNA intergenic spacer, partial sequence**

GAGTTCGCGGGAGAGTAAGTTATTGCCAGGGTTTTTATTTTATACTTTAAACATTGATTTTATTTTTTATGTTTTTAGATATTTATGTTTTTGAATGTTTTATTCAAATAATATAAAAAATAAAATATATATTGACATGGATTAAACAAAGATATATATTATTCTATGTTGTATAAACAAATTGGCAAAATAGAGATGGAAGATAAAAATATGGTCAAAGTAATAAGAGTCTATGGTGAATGCC

**>14_9_Borrelia garinii_5S-23S ribosomal RNA intergenic spacer, partial sequence**

GAGTTCGCGGGAGAGTAAGTTATTGCCAGGGTTTTTATTTTATACTTTAAACATTGATTTTATTTTTTATGTTTTTAGATGTTCATGTTTTTGAATGTTTTATTCGAATAATATAAAAAATAAAATATATATTGACATGGATTAAACAAAGATATATATTATTCTATGTTGTATAAACAAATTGGCAAAATAGAGATGGAAGATAAAAATATGGTCAAAGTAATAAGAGTCTATGGTGAATGCC

**>5_10_Borrelia garinii_5S-23S ribosomal RNA intergenic spacer, partial sequence**

GAGTTCGCGGGAGAGTAAGTTATTGCCAGGGTTTTTCTTTTATACTTTAAACATTGATTTTATTTTTTATGTTTTTAGATGTTTATATTATTTGAATGTTTTATTCAAATAATATAAAAAATAAAATATATATATTGACATGGATTAAACAAAGATATATATTATTCTATGTTGTATAAACAAATTGGCAAAATAGAGATGGAAGATAAAAATATGGTCAAAGTAATAAGAGTCTATGGTGAATGCC

**>9_11_Borrelia garinii_5S-23S ribosomal RNA intergenic spacer, partial sequence**

GAGTTCGCGGGAGAGTAAGTTGTTGCCAGGGTTTTTGTTTTATACTTTAAACATTGATTTTATTTTTTATGTTTTTAGATGTTCATGTTTTTGAATGTTTTATTCAAATAATATAAAAAATAAAATATATATTGACATGGATTAAACAAAGATATATATTATTCTATGTTGTATAAACAAATTGGCAAAATAGAGATGGAAGATAAAAATATGGTCAAAGTAATAAGAGTCTATGGTGAATGCC

**>10_11_Borrelia garinii_5S-23S ribosomal RNA intergenic spacer, partial sequence**

GAGTTCGCGGGAGAGTAAGTTGTTGCCAGGGTTTTTGTTTTATACTTTAAACATTGATTTTATTTTTTATGTTTTTAGATGTTCATGTTTTTGAATGTTTTATTCAAATAATATAAAAAATAAAATATATATTGACATGGATTAAACAAAGATATATATTATTCTATGTTGTATAAACAAATTGGCAAAATAGAGATGGAAGATAAAAATATGGTCAAAGTAATAAGAGTCTATGGTGAATGCC

**>10_12_Borrelia garinii_5S-23S ribosomal RNA intergenic spacer, partial sequence**

GAGTTCGCGGGAGAGTAAGTTATTGCCAGGGTTTTTATTTTATACTTTAAACATTGATTTTATTTTTTATGTTTTTAGATGTTCATGTTTTTGAATGTTTTATTCGAATAATATAAAAAATAAAATATATATTGACATGGATTAAACAAAGATATATATTATTCTATGTTGTATAAACAAATTGGCAAAATAGAGATGGAAGATAAAAATATGGTCAAAGTAATAAGAGTCTATGGTGAATGCC

**>33_13_Borrelia garinii_5S-23S ribosomal RNA intergenic spacer, partial sequence**

GAGTTCGCGGGAGAGTAAGTTATTGCCAGGGTTTTTATTTTATACTTTAAACATTGATTTTATTTTTTATGTTTTTAGATGTTCATGTTTTTGAATGTTTTATTCGAATAATATAAAAAATAAAATATATATTGACATGGATTAAACAAAGATATATATTATTCTATGTTGTATAAACAAATTGGCAAAATAGAGATGGAAGATAAAAATATGGTCAAAGTAATAAGAGTCTATGGTGAATGCC

**>24_16_Borrelia garinii_5S-23S ribosomal RNA intergenic spacer, partial sequence**

GAGTTCGCGGGAGAGTAAGTTATTGCCAGGGTTTTTATTTTATACTTTAAACATTGATTTTATTTTTTATGTTTTTAGATGTTCATGTTTTTGAATGTTTTATTCGAATAATATAAAAAATAAAATAATATTGACATGGATTAAACAAAGATATATATTATTCTATGTTGCATAAACAAATTGGCAAAATAGAGATGGAAGATAAAAATATGGTCAAAGTAATAAGAGTCTATGGTGAATGCC

**>33_16_Borrelia garinii_5S-23S ribosomal RNA intergenic spacer, partial sequence**

GAGTTCGCGGGAGAGTAAGTTATTGCCAGGGTTTTTATTTTATACTTTAAACATTGATTTTATTTTTTATGTTTTTAGATGTTCATGTTTTTGAATGTTTTATTCGAATAATATAAAAAATAAAATATATATTGACATGGATTAAACAAAGATATATATTATTCTATGTTGTATAAACAAATTGGCAAAATAGAGATGGAAGATAAAAATATGGTCAAAGTAATAAGAGTCTATGGTGAATGCC

**>12_17_Borrelia garinii_5S-23S ribosomal RNA intergenic spacer, partial sequence**

GAGTTCGCGGGAGAGTAAGTTATTGCCAGGGTTTTTATTTTATACTTTAAACATTGATTTTATTTTTTATGTTTTTAGATATTTATGTTTTTGAATGTTTTATTCAAATAATATAAAAAATAAAATATATATTGACATGGATTAAACAAAGATATATATTATTCTATGTTGTATAAACAAATTGGCAAAATAGAGATGGAAGATAAAAATATGGTCAAAGTAATAAGAGTCTATGGTGAATGCC

**>18_18_Borrelia garinii_5S-23S ribosomal RNA intergenic spacer, partial sequence**

GAGTTCGCGGGAGAGTAAGTTATTGCCAGGGTTTTTATTTTATACTTTAAACATTGATTTTATTTTTTATGTTTTTAGATGTTCATGTTTTTGAATGTTTTATTCGAATAATATAAAAAATAAAATATATATTGACATGGATTAAACAAAGATATATATTATTCTATGTTGTATAAACAAATTGGCAAAATAGAGATGGAAGATAAAAATATGGTCAAAGTAATAAGAGTCTATGGTGAATGCC

**>21_18_Borrelia garinii_5S-23S ribosomal RNA intergenic spacer, partial sequence**

GAGTTCGCGGGAGAGTAAGTTATTGCCAGGGTTTTTATTTTATACTTTAAACATTGATTTTATTTTTTATGTTTTTAGATGTTCATGTTTTTGAATGTTTTATTCGAATAATATAAAAAATAAAATATATATTGACATGGATTAAACAAAGATATATATTATTCTATGTTGTATAAACAAATTGGCAAAATAGAGATGGAAGATAAAAATATGGTCAAAGTAATAAGAGTCTATGGTGAATGCC

**>24_18_Borrelia garinii_5S-23S ribosomal RNA intergenic spacer, partial sequence**

GAGTTCGCGGGAGAGTAAGTTATTGCCAGGGTTTTTATTTTATACTTTAAACATTGATTTTATTTTTTATGTTTTTAGATGTTTATGTTTTTGAATGTTTTATTCAAATAATATAAAAAATAAAATATATATTGACATGGATTAAACAAAGATATATATTATTCTATGTTGTATAAACAAATTGGCAAAATAGAGATGGAAGATAAAAATATGGTCAAAGTAATAAGAGTCTATGGTGAATGCC

**>21_21_Borrelia garinii_5S-23S ribosomal RNA intergenic spacer, partial sequence**

GAGTTCGCGGGAGAGTAAGTTATTGCCAGGGTTTTTATTTTATACTTTAAACATTGATTTTATTTTTTATGTTTTTAGATGTTCATGTTTTTGAATGTTTTATTCGAATAATATAAAAAATAAAATATATATTGACATGGATTAAACAAAGATATATATTATTCTATGTTGTATAAACAAATTGGCAAAATAGAGATGGAAGATAAAAATATGGTCAAAGTAATAAGAGTCTATGGTGAATGCC

**>3_22_Borrelia garinii_5S-23S ribosomal RNA intergenic spacer, partial sequence**

GAGTTCGCGGGAGAGTAAGTTATTGCCAGGGTTTTTATTTTATACTTTAAACATTGATTTTATTTTTTATGTTTTTAGATGTTCATGTTTTTGAATGTTTTATTCGAATAATATAAAAAATAAAATATATATTGACATGGATTAAACAAAGATATATATTATTCTATGTTGTATAAACAAATTGGCAAAATAGAGATGGAAGATAAAAATATGGTCAAAGTAATAAGAGTCTATGGTGAATGCC

**>37_22_Borrelia garinii_5S-23S ribosomal RNA intergenic spacer, partial sequence**

GAGTTCGCGGGAGAGTAAGTTATTGCCAGGGTTTTTATTTTATACTTTAAACATTGATTTTATTTTTTATGTTTTTAGATGTTCATGTTTTTCAATGTTTTATTCAAATAATATAAAAAATAAAATATATATTGACATGGATTAAACAAAGATATATATTATTCTATGTTGTATAAACAAATTGGCAAAATAGAGATGGAAGATAAAAATATGGTCAAAGTAATAAGAGTCTATGGTGAATGCC

**>17_23_Borrelia garinii_5S-23S ribosomal RNA intergenic spacer, partial sequence**

GAGTTCGCGGGAGAGTAAGTTATTGCCAGGGTTTTTATTTTATACTTTAAACATTGATTTTATTTTTTATGTTTTTAGATGTTCATGTTTTTGAATGTTTTATTCGAATAATATAAAAAATAAAATATATATATTGACATGGATTAAACAAAGATATATATTATTCTATGTTGTATAAACAAATTGGCAAAATAGAGATGGAAGATAAAAATATGGTCAAAGTAATAAGAGTCTATGGTGAATGCC

**>18_23_Borrelia garinii_5S-23S ribosomal RNA intergenic spacer, partial sequence**

GAGTTCGCGGGAGAGTAAGTTATTGCCAGGGTTTTTATTTTATACTTTAAACATTGATTTTATTTTTTATGTTTTTAGATGTTCATGTTTTTGAATGTTTTATTCGAATAATATAAAAAATAAAATATATATTGACATGGATTAAACAAAGATATATATTATTCTATGTTGTATAAACAAATTGGCAAAATAGAGATGGAAGATAAAAATATGGTCAAAGTAATAAGAGTCTATGGTGAATGCC

**>19_23_Borrelia garinii_5S-23S ribosomal RNA intergenic spacer, partial sequence**

GAGTTCGCGGGAGAGTAAGTTATTGCCAGGGTTTTTATTTTATACTTTAAACATTGATTTTATTTTTTATGTTTTTAGATGTTCATGTTTTTGAATGTTTTATTCGAATAATATAAAAAATAAAATATATATATTGACATGGATTAAACAAAGATATATATTATTCTATGTTGTATAAACAAATTGGCAAAATAGAGATGGAAGATAAAAATATGGTCAAAGTAATAAGAGTCTATGGTGAATGCC

**>20_23_Borrelia garinii_5S-23S ribosomal RNA intergenic spacer, partial sequence**

GAGTTCGCGGGAGAGTAAGTTATTGCCAGGGTTTTTATTTTATACTTTAAACATTGATTTTATTTTTTATGTTTTTAGATGTTCATGTTTTTGAATGTTTTATTCGAATAATATAAAAAATAAAATATATATATTGACATGGATTAAACAAAGATATATATTATTCTATGTTGTATAAACAAATTGGCAAAATAGAGATGGAAGATAAAAATATGGTCAAAGTAATAAGAGTCTATGGTGAATGCC

**>29_23_Borrelia garinii_5S-23S ribosomal RNA intergenic spacer, partial sequence**

GAGTTCGCGGGAGAGTAAGTTATTGCCAGGGTTTTTATTTTATACTTTAAACATTGATTTTATTTTTTATGTTTTTAGATGTTTATGTTTTTGAATGTTTTATTCAAATAATATAAAAAATAAAATATATATTGACATGGATTAAACAAAGATATATATTATTCTATGTTGTATAAACAAATTGGCAAAATAGAGATGGAAGATAAAAATATGGTCAAAGTAATAAGAGTCTATGGTGAATGCC

**>30_23_Borrelia garinii_5S-23S ribosomal RNA intergenic spacer, partial sequence**

GAGTTCGCGGGAGAGTAAGTTATTGCCAGGGTTTTTATTTTATACTTTAAACATTGATTTTATTTTTTATGTTTTTAGATGTTTATGTTTTTGAATGTTTTATTCAAATAATATAAAAAATAAAATATATATTGACATGGATTAAACAAAGATATATATTATTCTATGTTGTATAAACAAATTGGCAAAATAGAGATGGAAGATAAAAATATGGTCAAAGTAATAAGAGTCTATGGTGAATGCC

**>31_23_Borrelia garinii_5S-23S ribosomal RNA intergenic spacer, partial sequence**

GAGTTCGCGGGAGAGTAAGTTATTGCCAGGGTTTTTATTTTATACTTTAAACATTGATTTTATTTTTTATGTTTTTAGATGTTTATGTTTTTGAATGTTTTATTCAAATAATATAAAAAATAAAATATATATTGACATGGATTAAACAAAGATATATATTATTCTATGTTGTATAAACAAATTGGCAAAATAGAGATGGAAGATAAAAATATGGTCAAAGTAATAAGAGTCTATGGTGAATGCC

**>43_23_Borrelia garinii_5S-23S ribosomal RNA intergenic spacer, partial sequence**

GAGTTCGCGGGAGAGTAAGTTATTGCCAGGGTTTTTATTTTATACTTTAAACATTGATTTTATTTTTTATATTATTTGAATAAAACATTCAAATAATATAAAAAATAAAATATATATTGACATGGATTAAACAAAGATATATATTATTCTATGTTGTATAAACAAATTGGCAAAATAGAGATGGAAGATAAAAATATGGTCAAAGTAATAAGAGTCTATGGTGAATGCC

**>31_24_Borrelia garinii_5S-23S ribosomal RNA intergenic spacer, partial sequence**

GAGTTCGCGGGAGAGTAAGTTATTGCCAGGGTTTTTATTTTATACTTTAAACATTGATTTTATTTTTTATATTATTTGAATAAAACATTCAAATAATATAAAAAATAAAATATATATTGACATGGATTAAACAAAGATATATATTATTCTATGTTGTATAAACAAATTGGCAAAATAGAGATGGAAGATAAAAATATGGTCAAAGTAATAAGAGTCTATGGTGAATGCC

**>33_24_Borrelia garinii_5S-23S ribosomal RNA intergenic spacer, partial sequence**

GAGTTCGCGGGAGAGTAAGTTATTGCCAGGGTTTTTATTTTATACTTTAAACATTGATTTTATTTTTTATATTATTTGAATAAAACATTCAAATAATATAAAAAATAAAATATATATTGACATGGATTAAACAAAGATATATATTATTCTATGTTGTATAAACAAATTGGCAAAATAGAGATGGAAGATAAAAATATGGTCAAAGTAATAAGAGTCTATGGTGAATGCC

**>35_24_Borrelia garinii_5S-23S ribosomal RNA intergenic spacer, partial sequence**

GAGTTCGCGGGAGAGTAAGTTGTTGCCAGGGTTTTTGTTTTATACTTTAAACATTGATTTTATTTTTTATGTTTTTAGATGTTCATGTTTTTGAATGTTTTATTCAAATAATATAAAAAATAAAATATATATTGACATGGATTAAACAAAGATATATATTATTCTATGTTGTATAAACAAATTGGCAAAATAGAGATGGAAGATAAAAATATGGTCAAAGTAATAAGAGTCTATGGTGAATGCC

**>35_25_Borrelia garinii_5S-23S ribosomal RNA intergenic spacer, partial sequence**

GAGTTCGCGGGAGAGTAAGTTATTGCCAGGGTTTTTATTTTATACTTTAAACATTGATTTTATTTTTTATGTTTTTAGATGTTCATGTTTTTCAATGTTTTATTCAAATAATATAAAAAATAAAATATATATTGACATGGATTAAACAAAGATATATATTATTCTATGTTGTATAAACAAATTGGCAAAATAGAGATGGAAGATAAAAATATGGTCAAAGTAATAAGAGTCTATGGTGAATGCC

**>36_25_Borrelia garinii_5S-23S ribosomal RNA intergenic spacer, partial sequence**

GAGTTCGCGGGAGAGTAAGTTATTGCCAGGGTTTTTATTTTATACTTTAAACATTGATTTTATTTTTTATGTTTTTAGATGTTCATGTTTTTCAATGTTTTATTCAAATAATATAAAAAATAAAATATATATTGACATGGATTAAACAAAGATATATATTATTCTATGTTGTATAAACAAATTGGCAAAATAGAGATGGAAGATAAAAATATGGTCAAAGTAATAAGAGTCTATGGTGAATGCC

**>45_25_Borrelia garinii_5S-23S ribosomal RNA intergenic spacer, partial sequence**

GAGTTCGCGGGAGAGTAAGTTATTGCCAGGGTTTTTATTTTATACTTTAAACATTGATTTTATTTTTTATGTTTTTAGATGTTCATGTTTTTGAATGTTTTATTCAAATAATATAAAAAATAAAATATATATTGACATGGATTAAACAAAGATATATATTATTCTATGTTGTATAAACAAATTGGCAAAATAGAGATGGAAGATAAAAATATGGTCAAAGTAATAAGAGTCTATGGTGAATGCC

**>45_26_Borrelia garinii_5S-23S ribosomal RNA intergenic spacer, partial sequence**

GAGTTCGCGGGAGAGTAAGTTATTGCCAGGGTTTTTATTTTATACTTAAACATTGATTTTATTTTTTATGTTTTTAGATATTTATGTTTTTGAATGTTTTATTCAAATAATATAAAAAATAAAATATATATTGACATGGATTAAACAAAGATATATATTATTCTATGTTGTATAAACAAATTGGCAAAATAGAGATGGAAGATAAAAATATGGTCAAAGTAATAAGAGTCTATGGTGAATGCC

**>29_27_Borrelia garinii_5S-23S ribosomal RNA intergenic spacer, partial sequence**

GAGTTCGCGGGAGAGTAAGTTATTGCCAGGGTTTTTATTTTATACTTTAAACATTGATTTTATTTTTTATGTTTTTAGATATTTATGTTTTTGAATGTTTTATTCAAATAATATAAAAAATAAAATATATATTGACATGGATTAAACAAAGATATATATTATTCTATGTTGTATAAACAAATTGGCAAAATAGAGATGGAAGATAAAAATATGGTCAAAGTAATAAGAGTCTATGGTGAATGCC
